# A humanized monoclonal antibody against the endothelial chemokine CCL21 for the diagnosis and treatment of inflammatory bowel disease

**DOI:** 10.1371/journal.pone.0252805

**Published:** 2021-07-01

**Authors:** Maegan L. Capitano, Aruna Jaiswal, Hal E. Broxmeyer, Yilianys Pride, Sarah Glover, Fatemah G. Amlashi, Austin Kirby, Gayathri Srinivasan, Elizabeth A. Williamson, Daniel Mais, Robert Hromas

**Affiliations:** 1 Department of Microbiology and Immunology, Indiana University School of Medicine, Indianapolis, IN, United States of America; 2 Department of Medicine and the Mays Cancer Center, University of Texas School Health Science Center at San Antonio, San Antonio, TX, United States of America; 3 Division of Gastroenterology, Department of Medicine, University of Mississippi Medical Center, Jackson, MI, United States of America; 4 Department of Pathology, University of Texas School Health Science Center at San Antonio, San Antonio, TX, United States of America; Institut national de la santé et de la recherche médicale: INSERM, FRANCE

## Abstract

Chemokines are small proteins that promote leukocyte migration during development, infection, and inflammation. We and others isolated the unique chemokine CCL21, a potent chemo-attractant for naïve T-cells, naïve B-cells, and immature dendritic cells. CCL21 has a 37 amino acid carboxy terminal extension that is distinct from the rest of the chemokine family, which is thought to anchor it to venule endothelium where the amino terminus can interact with its cognate receptor, CCR7. We and others have reported that venule endothelium expressing CCL21 plays a crucial role in attracting naïve immune cells to sites of antigen presentation. In this study we generated a series of monoclonal antibodies to the amino terminus of CCL21 in an attempt to generate an antibody that blocked the interaction of CCL21 with its receptor CCR7. We found one humanized clone that blocked naïve T-cell migration towards CCL21, while memory effector T-cells were less affected. Using this monoclonal antibody, we also demonstrated that CCL21 is expressed in the mucosal venule endothelium of the large majority of inflammatory bowel diseases (IBD), including Crohn’s disease, ulcerative colitis, and also in celiac disease. This expression correlated with active IBD in 5 of 6 cases, whereas none of 6 normal bowel biopsies had CCL21 expression. This study raises the possibility that this monoclonal antibody could be used to diagnose initial or recurrent of IBD. Significantly, this antibody could also be used for therapeutic intervention in IBD by selectively interfering with recruitment of naïve immune effector cells to sites of antigen presentation, without harming overall memory immunity.

## Introduction

Chemokines are a family of small proteins that share structural and functional elements. They share cysteine-mediated covalent bonds in the amino terminus and mediation of leukocyte migration [1, 2]. Chemokines and their receptors are important in many types of human diseases, playing crucial roles in inflammatory tissue destruction seen in atherosclerosis, adult respiratory distress syndrome, cerebral vascular events, and myocardial infarction [3–5]. We also previously demonstrated that chemokines can mediate the inhibition of hematopoiesis seen during systemic inflammation [6]. In addition, chemokines can promote the aberrant migration of leukocytes into target organs during autoimmune diseases, such as lupus, rheumatoid arthritis, and inflammatory bowel disease [5, 7, 8].

We and others cloned and characterized a novel CC chemokine designated CCL21 that is a potent chemo-attractant for naïve T-cells and immature dendritic cells [9–11]. CCL21 directed migration of naive B-cells and natural killer (NK) cells, although to a lesser extent than T-cells [9–11]. It does not direct migration of monocytes or granulocytes [9–11]. CCL21 also promotes naïve lymphocyte adhesion to the endothelium of small venules, most notably in lymph nodes where naïve lymphocytes can be presented with antigen, but also in primary inflamed tissues as well [12–14]. The expression of CCL21 in the high endothelial venules of lymph nodes therefore mediates naïve T-cell trafficking to secondary lymphoid organs for antigen presentation.

We previously reported that in skin autoimmune disease, CCL21 was highly induced in the venule endothelium of the inflamed tissue but not in normal tissue [15]. In addition, the vast majority of the infiltrating T-cells expressed CCR7, the receptor for CCL21. This raised the possibility that local auto-antigen presentation in the primary inflamed tissue could be more important than secondary lymphoid tissue auto-antigen presentation in generating local tissue damaging effector T-cells [12–15].

While progress has been made, changing the natural history of inflammatory bowel diseases (IBD) is still problematic because the initiators of the disease are imperfectly defined [16, 17]. The goal of interrupting the signaling cascade that leads to the destruction of the intestinal mucosa in these diseases has been hampered not just by the lack of targets but by the ineffectiveness of therapies against the targets that are known [5, 8, 16–19]. Since the gut is its own secondary lymphoid organ where naïve lymphocytes can be presented antigen locally instead of maturing in a secondary lymphoid organ and then migrating to target inflamed/infected tissues, one potential IBD therapeutic target could be interrupting the migration of naïve lymphoid cells into the inflamed gut [5, 7, 8, 16]. CCL21 expression correlated with induction of ulcerative colitis in mice, and when that colitis was treated CCL21 expression decreased [8, 18]. Therefore, we hypothesized that blocking CCL21-directed migration of naïve immune cells might alter the natural history of IBD [19, 20]. While there is at least one commercially available neutralizing monoclonal antibody against human CCL21 (R&D Systems, Minneapolis, MN), its activity in immunohistology of endothelial CCL21 in T-cell autoimmune diseases and its activity against Th-cell subsets has not been characterized. In addition, this has not been humanized and thus would not be appropriate for clinical development as a therapeutic agent in T-cell autoimmune diseases.

To bring this concept closer to clinical testing, we generated a series of monoclonal antibodies to the amino terminus of human CCL21 [21] and found one clone that completely blocked naïve T-cell migration towards CCL21. However, migration of memory T-cell towards CCL21 was less affected. Using this monoclonal antibody, we also demonstrated that CCL21 is expressed in the mucosal venule endothelium of the majority of inflammatory bowel diseases, including Crohn’s disease, ulcerative colitis, and celiac disease. This raises the possibility that this monoclonal antibody could serve as a diagnostic marker of active IBD, and also prevent initiation or recurrence of inflammatory bowel diseases by selectively interfering with recruitment of naïve immune effector cells to sites of antigen presentation, without harming overall memory immunity [16–19].

## Materials and methods

### Monoclonal antibody generation

The mouse facility satisfies the most stringent and modern international regulations on animal rights and protection. Mice were kept in autoclaved, ventilated micro-isolator cages with free access to water and standard mouse chow (University of Florida IACUC reviewed and approved the Study#201810293). The facility follows NIH guidelines for the care and use of animals and has certified veterinarians on-site for oversight and consultation. The animal care program was under the direction of 4 full-time ACLAM-certified laboratory animal veterinarians. At least one veterinarian is available 24 hours a day for routine as well as emergency care. Two BALB/C mice (Jackson Labs, Bar Harbor, ME) were subcutaneously immunized with 2 mg each of human CCL21 recombinant full-length protein in complete Freund’s adjuvant and boosted three times with the equivalent preparation before splenic harvest (University of Florida IACUC reviewed and approved Study #201609334). Euthanasia was performed by CO_2_ inhalation followed by cervical dislocation and splenocytes harvested in a sterile manner. We screened 196 fused plasma cell clones that by ELISA produced an antibody that reacted with human CCL21 for binding to the amino terminus of human CCL21 protein. We tested whether two peptides from the amino terminus of hCCL21, which interacts with CCR7 could compete off the antibody clone binding to CCL21 using slot blot. Full length recombinant mouse CCL21 protein was purchased from R&D Systems. Full length recombinant human CCL21 protein was purchased from Novus Biologicals, Centennial, CO, USA. CCL21 (8–20 aa) and CCL21 (43–56 aa) peptides were synthesized by New England Peptide (Gardner, MA, USA). When the best performing murine clone was identified, it was humanized via gene conversion using preassembled oligonucleotide mutagenesis [22, 23]. Sixteen human IgG1-LALA clones were generated that had varied characteristics for solubility, isomerization, glycosylation, free cysteines, or deamidation. None could fix complement.

### Human peripheral blood T-cell isolation

Normal donor human peripheral blood (PB) was collected in accordance to Indiana University School of Medicine’s Institutional Review Board (Indiana University IRB reviewed and approved the Study #1011002987). Volunteer donors were recruited by public notice, signed an informed consent, and donated blood in the first author’s laboratory in a designated area under sterile precautions. Mononuclear cell layer was collected using Ficoll-Paque PLUS (GE Healthcare Bio-Sciences AB; Pittsburgh, PA) density gradient centrifugation. The CD3+ PB cells were then isolated using immunoaffinity selection with MiniMACS paramagnetic CD3 microbeads (Miltenyi Biotec; Auburn, CA) using two sequential LS columns (Miltenyi Biotec, Auburn, CA).

### T-cell chemotaxis assays

T-cell chemotaxis was measured as previously described [9, 20]. Human PB T-cells acclimated to 37°C were suspended in prewarmed RPMI (37°C) with 0.5% bovine serum albumin (BSA; Sigma-Aldrich; St. Louis, MO). Costar 24-well transwell plates with 6.5 mm diameter inserts with 5.0 μm pores (Sigma-Aldrich, St. Louis, MO) were prepared by placing 650 μl of prewarmed RPMI with 0.5% BSA that contained 0, 1200 ng/mL rhCCL21 (R&D Systems, Minneapolis, MN) or 1200ng/mL rhCCL21 pretreated for 1 hour with sample clones #7–39 in the bottom well and allowing plates to acclimate at 37°C for half an hour prior to chemotaxis assay. Cells were suspended at 300,000 cells/100 μl prewarmed RPMI with 0.5% BSA and loaded to the top chamber of the transwell assay. Transwell plates were placed in a 37°C incubator (95% humidity, 5% CO2) for 4 hours. Percent migration was determined using flow cytometry (counts determined by running samples for the same amount of time at the same speed) with background migration (cells that migrated toward media alone; always <4%) subtracted from total migrated cells.

### Flow cytometry

To analyze which T cell populations which migrated in our chemotaxis assays [20], after counting by flow (see above), cells were washed in PBS, incubated in fluorescently conjugated anti-human antibody cocktail for 20 minutes at room temperature, washed in PBS, and then fixed in 1% formaldehyde. The samples were analyzed on an LSR II flow cytometer (BD Biosciences; San Jose, CA). Antibody concentrations were used as stated by manufacturer’s instructions. Data analysis was performed using FlowJo 7.6.3 software (Tree Star; Ashland, OR). Gates were determined using fluorescence minus one control. The following markers were used: APC-H7 conjugated anti-human CD3 (clone SK7), PerCP-Cy5.5 conjugated anti-human CD4 (clone SK3), FITC conjugated anti-human CD45RA (clone HI100), Alexa Fluor® 647 conjugated anti-human CD197 (CCR7; clone 150503) (components of a Human Naïve/Memory T cell Panel Kit from BD Biosciences cat. # 561438; San Jose, CA, USA), BV421 conjugated anti-human CD8 (clone RPA-T8; BD Biosciences, San Jose, CA), PE conjugated anti-human CD27 (clone M-T271; BD Biosciences; San Jose, CA). For all antibodies used in these studies, the validation for the relevant species and applications can be found on the indicated manufacturer’s website.

### Immunohistology

Immunohistology was performed as previously described [15]. Under IRB approval (University of Mississippi IRB reviewed and approved Protocols 2019–0089 and 2019–0081), the University of Mississippi Medical Center anatomic pathology database was searched for cases of psoriasis (positive controls), inflammatory bowel disease (Crohn’s disease and ulcerative colitis), celiac sprue, and rheumatoid arthritis (negative controls). Samples were anonymized before sending for immunohistology. Hematoxylin and eosin-stained slides were reviewed to confirm diagnoses in order to select blocks for immunohistochemical staining. For all cases, 4-micron thick sections were cut from the paraffin-embedded formalin-fixed tissue blocks and placed on charged slides. Sections were deparaffinized in xylene and rehydrated through graded alcohols to distilled water before undergoing antigen retrieval. Immunohistochemistry was performed using the Discovery Ultra automated instrument (Roche; Indianapolis, IN, USA) per the manufacturer’s instructions, with the CCL21 antibody clone 8 at a 1:1000 dilution, and anti-mouse conjugated with DAB. Positive controls of normal human lymph node and negative controls of like tissue with mouse IgG were also performed. Immunoreactivity was qualitatively assessed by 2 pathologists. Counter-staining was performed with hematoxylin and eosin. Reactions were judged negative if there was no capillary endothelial expression, only rare capillaries expressing, or weak or blush discontinuous expression. Staining was read blind to diagnoses, and classified as positive if there was multifocal, strong, and nearly circumferential capillary endothelial expression.

## Results and discussion

### Generation of monoclonal antibodies against the amino terminus of human CCL21

**Fig 1:**
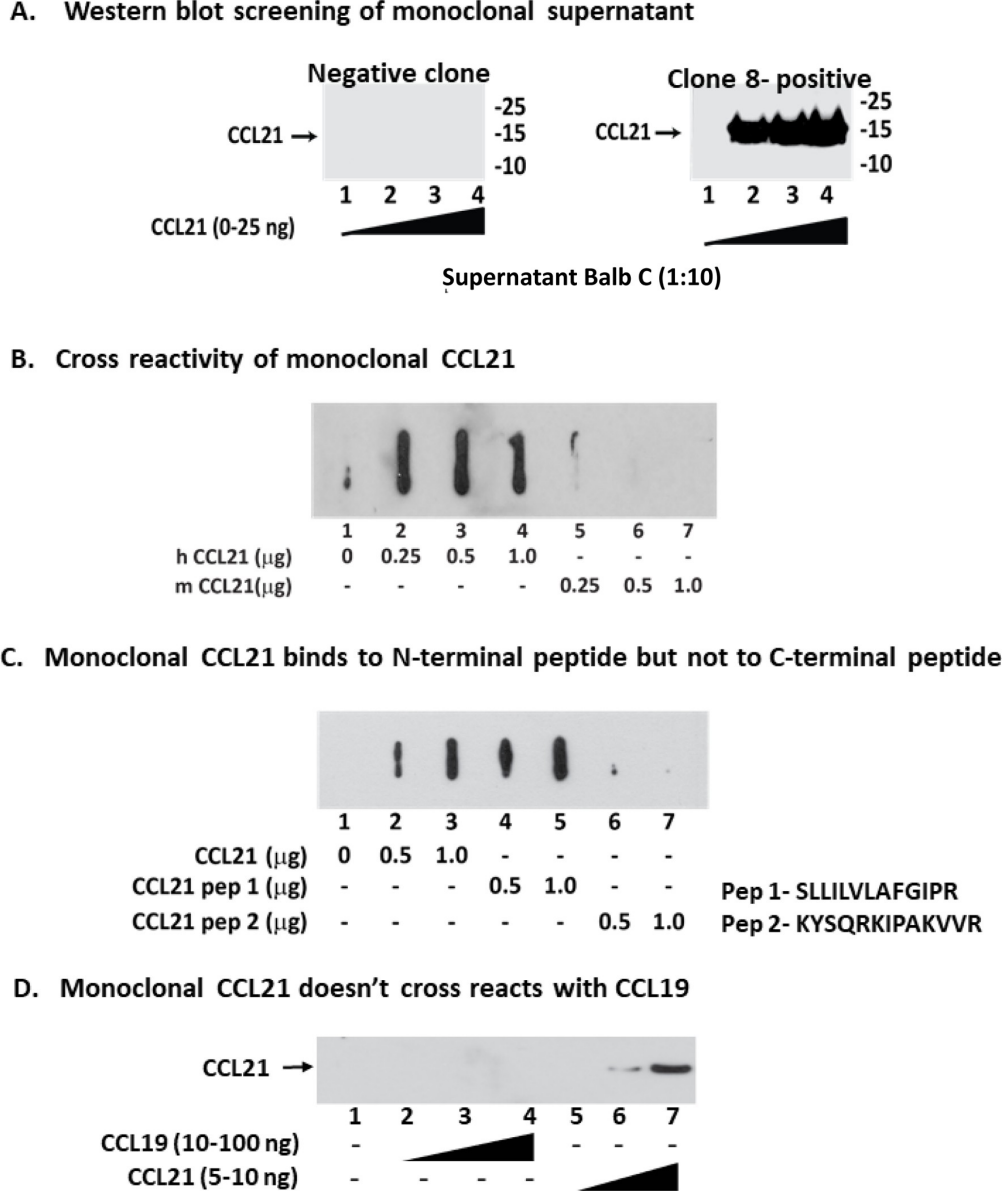
Screening monoclonal antibody clones against the amino terminus of CCL21. **A)** Screening of CCL21 monoclonal antibody clones by western blotting for binding to the human CCL21 protein as a single band. Clone #8 (C8) results are shown for all assays in this figure. **B)** Cross reactivity of monoclonal anti-CCL21 antibody to human and mouse CCL21 protein. We then screened the clones for lack of binding to murine CCL21 by slot blot hybridization. **C)** Screening of a peptide from the CCR7-interacting amino terminal region of CCL21 could compete off the monoclonal from binding CCL21 using slot blot hybridization. **D)** Screening of monoclonal antibody clones for lack of binding to the related human chemokine CCL19. Thirty-three monoclonal antibodies passed all of these screens.

The amino terminus of CCL21 is the region that interacts with its receptor, CCR7 [21]. We subcutaneously immunized BALB/C mice with human CCL21 recombinant full-length protein in adjuvant and boosted three times before splenic harvest. We screened 196 fused plasma cell clones that by ELISA produced an antibody that reacted with human CCL21 for western blot recognition of human CCL21 protein as a single band (Figs 1A and S1A). Using slot blot analysis, we then screened for titer and characterized the specificity of these antibodies (Figs 1B and S1B). The amino terminus of CCL21 is the protein region that interacts with its receptor CCR7, while carboxy terminus is thought to interact with endothelial extracellular heparinoids, anchoring the chemokine to the endothelial cell surface [14, 15, 21]. Screening was then performed by assessing whether peptides homologous to the amino terminus of CCL21 could compete off the monoclonal antibody in the slot blot analysis. We used peptides containing amino acids 8–20 and 43–56 from the amino terminal CCR7-interacting regions of CCL21 to compete against the monoclonal antibody clones binding to CCL21 (Figs 1C and S1C and S1D) [21]. High titer antibodies were also assessed by western analysis for specific binding to human CCL21 and not the related chemokine CCL19 (Fig 1D). After these screens were complete, we had 33 clones left for analysis that selectively bound to the amino terminus CCL21 at a high titer. These clones were then assessed for inhibition of T-cell migration in chemotaxis assays.

### Monoclonal antibody blockade of human CCL21 function

**Fig 2:**
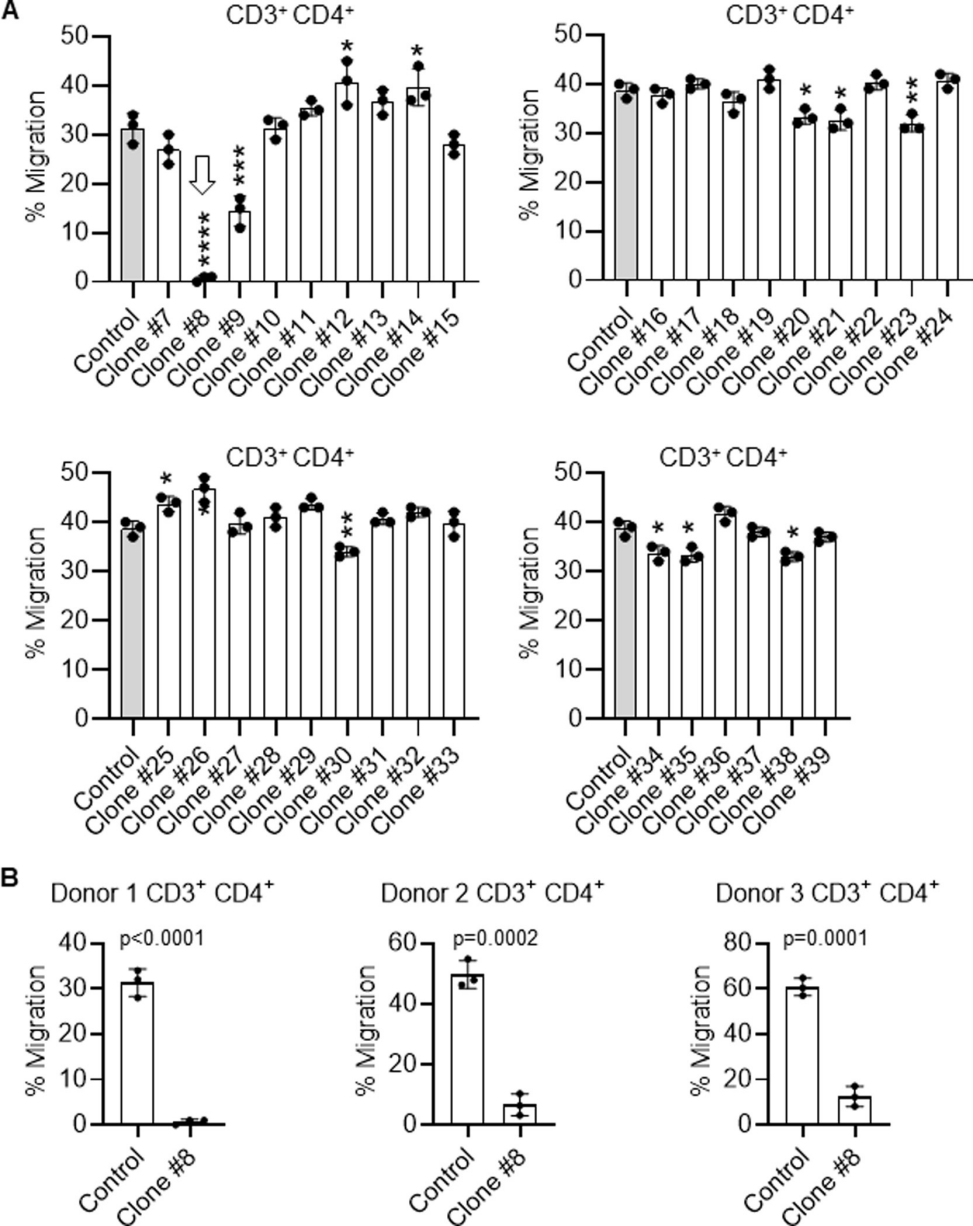
Screening of monoclonal antibody clones against CCL21-mediated T-cell chemotaxis. **A)** Inhibition of migration in transwell chemotaxis assays of human helper T-cells (CD3^+^/CD4^+^) towards human CCL21 by the 33 murine monoclonal antibody clones that passed the screening process outlined in Fig 1. Clone 8 (C8) completely abrogated T-cell migration towards CCL21, while clone 9 partially inhibited migration. Several clones promoted migration of T-cells, suggesting they enhanced CCL21 presentation to its receptor, CCR7. **B)** C8 also blocked migration of three distinct normal donor human Th-cells towards CCL21, indicating this is a general phenomenon. For all migration assays, data are the mean ± SD of triplicate wells, performed twice. Student T tests were performed for statistical analysis for this figure and Fig 3. * p<0.05, ** p<0.01, *** p<0.001 and **** p<0.0001 when compared to control.

Using transwell chemotaxis assays we measured the ability of each of the 33 monoclonal antibody clones to block CD3^+^/CD4^+^ helper (h) T-cell migration from the top well to the lower well in response to a CCL21 gradient (Figs 2 and S2–S6). We found one clone (Clone #8, hereafter C8) that completely abrogated Th-cell chemotaxis towards CCL21 and one that partially blocked Th-cell chemotaxis towards CCL21 (Clone #9). There were no other clones that had any significant inhibitory activity. Interestingly, clones #12–14, 25, 26, each enhanced chemotaxis towards CCL21 (Fig 2A), implying that they improved presentation of CCL21 to its receptor CCR7 on Th-cells. We repeated the Th-cell chemotaxis inhibition assays with C8 on three total normal donors and found that C8 inhibited CCL21-directed migration for all three normal Th-cell samples, indicating that this was a general finding, and not individualized.

### CCL21 promotes migration of naive helper T-cells but not memory T-cells

**Fig 3:**
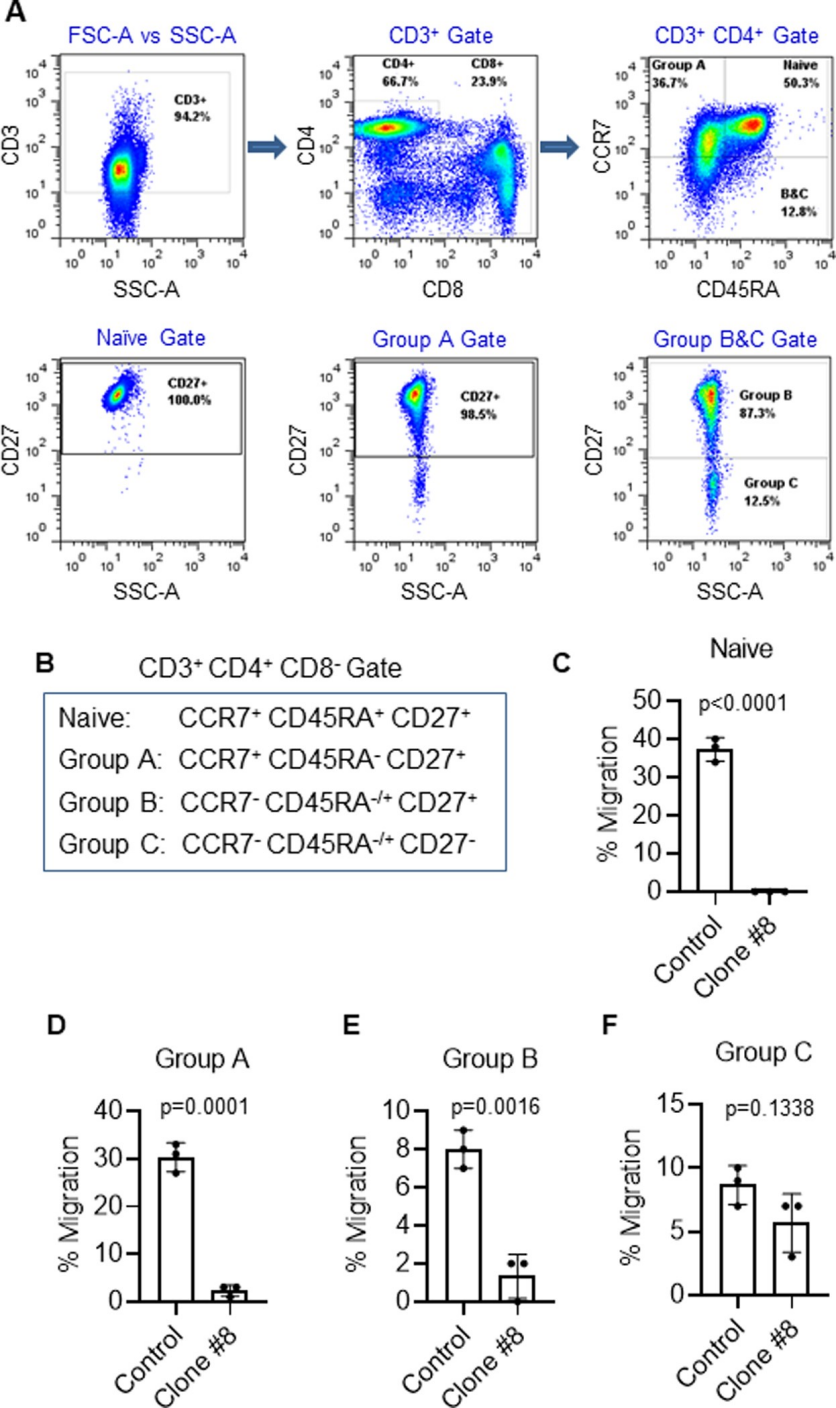
Inhibition of T-cell subset migration towards CCL21 by C8. **(A)** Flow cytometric gating strategy for isolation of human Th-cell subpopulations for testing the effects of C8 on migration towards CCL21. Migration of defined populations of T cell subsets was determined using flow cytometry of the lower versus upper chambers in transwell chemotaxis assays with background migration (cells that migrated toward media with no chemokine) subtracted from total cells. **(B)** Surface biomarker identification of Th-cell subsets tested in these experiments. **(C-F)** Fractional migration towards CCL21 inhibited by C8 in these T-cell subsets. Naïve Th-cells had the greatest migration towards CCL21 and were the most inhibited. Effect of C8 on migration towards CCL21 decreases as Th-cells become more mature.

Using flow cytometric analysis of T-cell subsets, we assessed the type of T-cell that responds to CCL21 and were blocked by anti-CCL21 C8. CD45RA and CD27 expression define naïve T-cells which have not yet been exposed to or responded to antigen (Fig 3A and 3B) [20]. We found that CD3^+^/CD4^+^/CD45RA^+^/CD27^+^ naïve Th-cells migrated best to CCL21 and that this migration was nearly completely blocked by C8 (Figs 3C and S7). However, Th-cells that do not express CD27, which are cells that may have already responded to antigen, migrate with significantly less frequency to CCL21, and C8 was less successful in inhibited migration (Fig 3D–3F). CCL21 has a minor non-canonical receptor CCRL1 that can be responsible for residual migration towards CCL21 in CCR7^-^ T-cells (Fig 3E and 3F) [24].

### Immunohistologic analysis of the expression of CCL21 in the endothelium of intestinal autoimmune diseases

**Fig 4:**
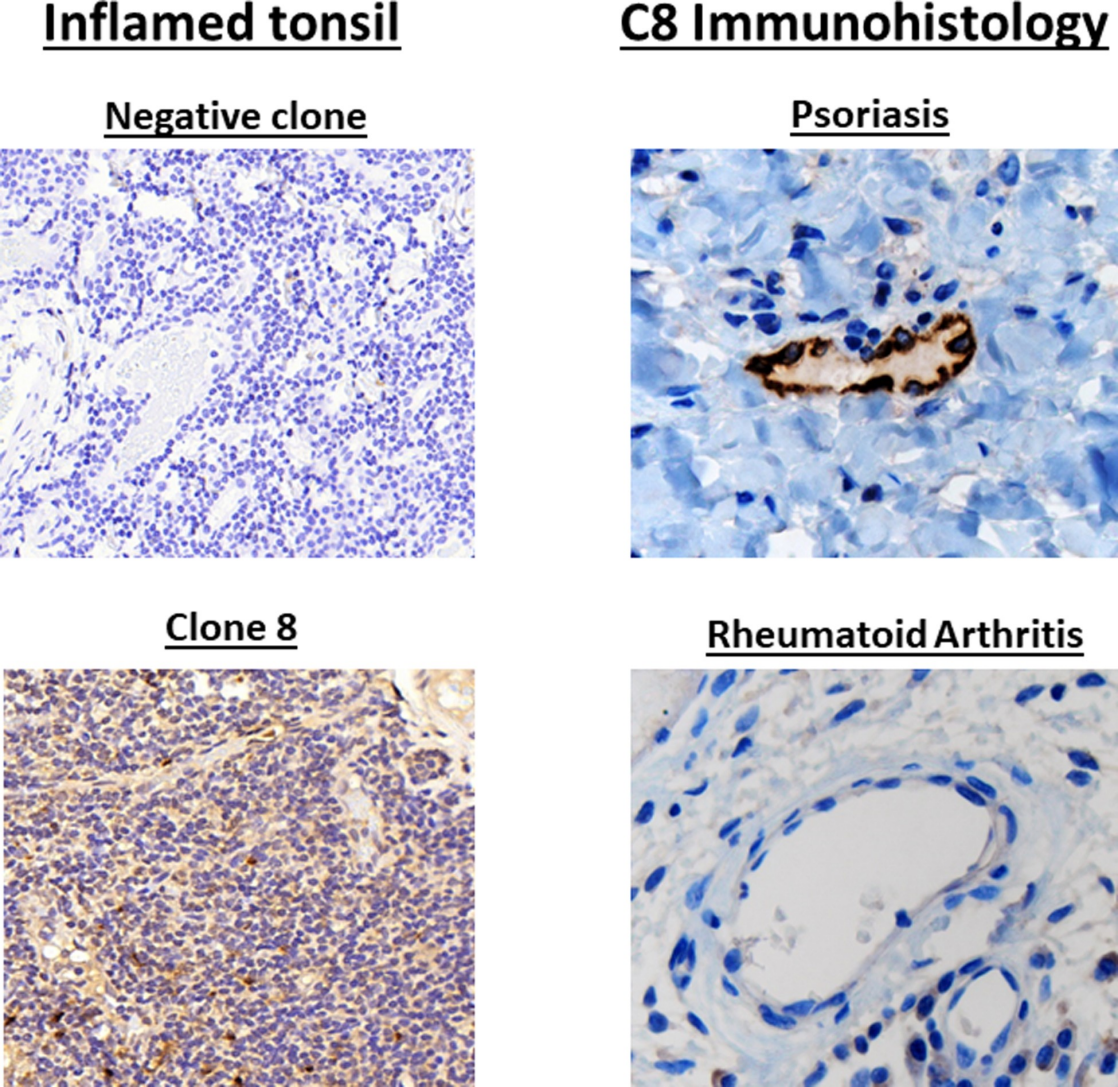
Screening of C8 for immunohistologic recognition of CCL21 in a tonsilar lymphoid biopsy specimen. Screening for binding of CCL21 in human lymphoid tissue. C8 was positive for binding to appropriate CCL21-expressing cells in human tonsil lymph node, while another monoclonal antibody in this series that failed screening in Fig 1 did not bind to any cells, indicating the utility of C8 as an immunohistologic tool (left panels). CCL21 is expressed in the venule endothelium of T-cell-mediated auto-immune skin diseases [15]. C8 specifically recognizes this expression in immunohistologic assays while it did not recognize synovium from rheumatoid arthritis serves as a negative control, as it is mediated by a distinct immunologic pathway (right panels).

We first tested whether C8 and another murine monoclonal antibody among the original cohort that failed to bind to human CCL21 bound to appropriate cells in a human tonsil lymph node (Fig 4, left upper panel). We found that C8 bound to appropriate cells in the lymph node- dendritic cells, endothelial cells and lymphocytes whereas the negative control monoclonal antibody did not (Fig 4, left lower panel). We also tested whether C8 bound specifically to the endothelium of active psoriasis as a positive control, since we had previously published that T-cell-infiltrative autoimmune diseases of the skin expressed CCL21 in the venule endothelium [15]. There was CCL21 expression in the dermal venule endothelium recognized by C8 in 6 of 8 cases of psoriasis, the positive controls (Fig 4, right upper panel) [15]. We used rheumatoid arthritis as a negative control since T-cell infiltration in this autoimmune disease is mediated by a distinct mechanism [7]. C8 did not bind to the endothelium of any of the 6 cases of synovium in rheumatoid arthritis (Fig 4, right lower panel).

**Fig 5:**
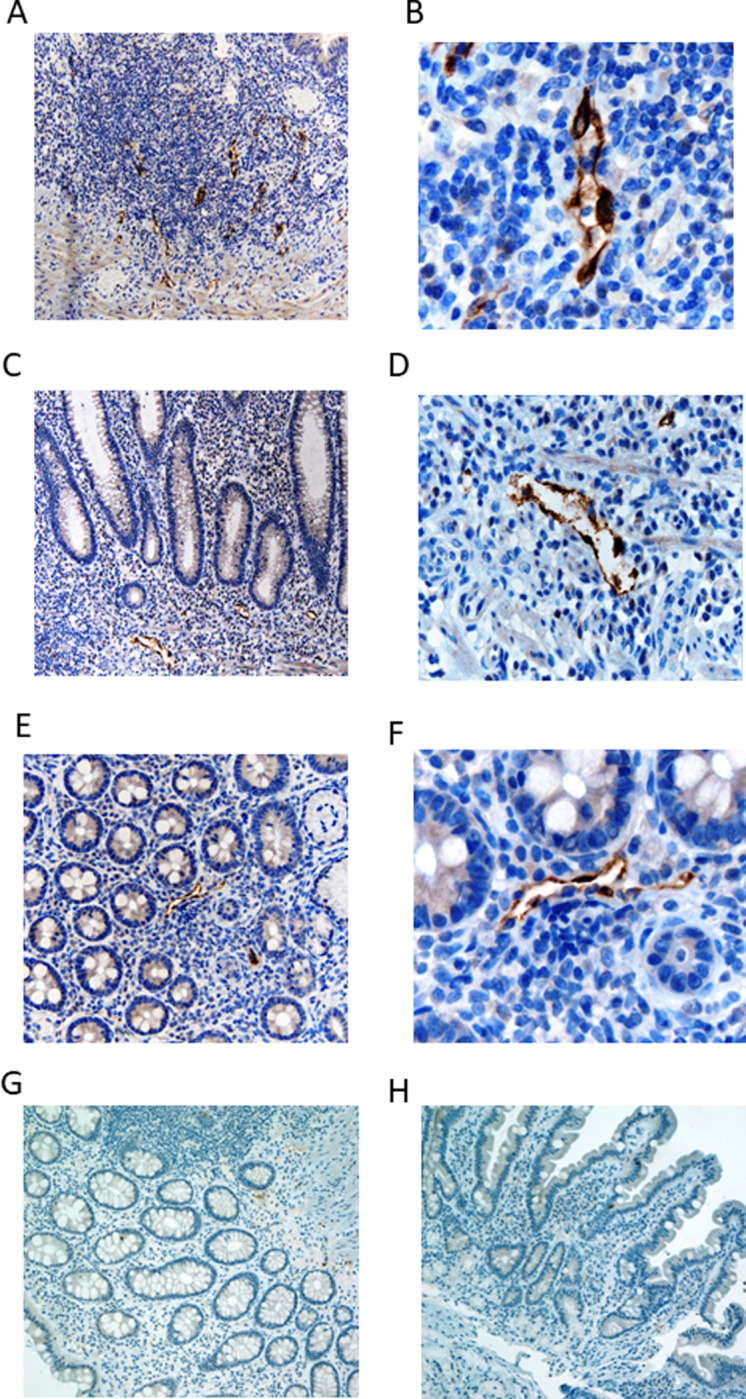
Immunohistology of venule endothelial expression of CCL21 in intestinal mucosal autoimmune diseases. **A)** 40x magnification of 1 of 3 Crohn’s disease intestinal mucosa biopsy immunohistologically stained with the C8 (anti-CCL21 monoclonal antibody). Two of 3 had similar positive results as shown here. **B)** 100 x magnification of (A). **C)** 40x magnification of 1 of 3 ulcerative colitis colonic mucosa biopsy immunohistologically stained with C8. All 3 biopsies had results nearly identical to that show here. **D)** 100 x magnification of (C). **E)** 40x magnification of 1 of 6 colonic mucosal biopsies of celiac sprue disease immunohistologically stained with C8. Four of the 6 samples had nearly identical results with that shown here. **F)** 100 x magnification of (E). **(G)** Normal duodenal endothelium does not express CCL21. One of 3 biopsy samples shown here that is representative of all 3. **H)** Normal colonic endothelium does not express CCL21. One representative sample of 3 biopsies shown here.

We next examined the binding of C8 in biopsy specimens of intestinal autoimmune diseases (Crohn’s disease, ulcerative colitis, and celiac sprue disease) using immunohistology (Figs 5 and S8). There was CCL21 expression in the submucosal venule endothelium recognized by C8 in 2 of 3 cases of Crohn’s disease (Fig 5A and 5B), in 3 of 3 cases of ulcerative colitis (Fig 5C and 5D), and in 4 of 6 cases of sprue (Fig 5E and 5F). There was no expression of CCL21 in 3 normal duodenal biopsies (Fig 5G) or in 3 normal colonic biopsy specimens (Fig 5H). Using chi square analysis for comparison of CCL21 endothelial expression in all of the IBD samples with the normal bowel samples, the 5 positives in the 6 IBD samples have a p value of 0.049 for being different from the 0 of 6 positives in the normal bowel specimens.

### Humanization of C8 maintained inhibition of naïve T-cell migration towards CCL21

**Fig 6:**
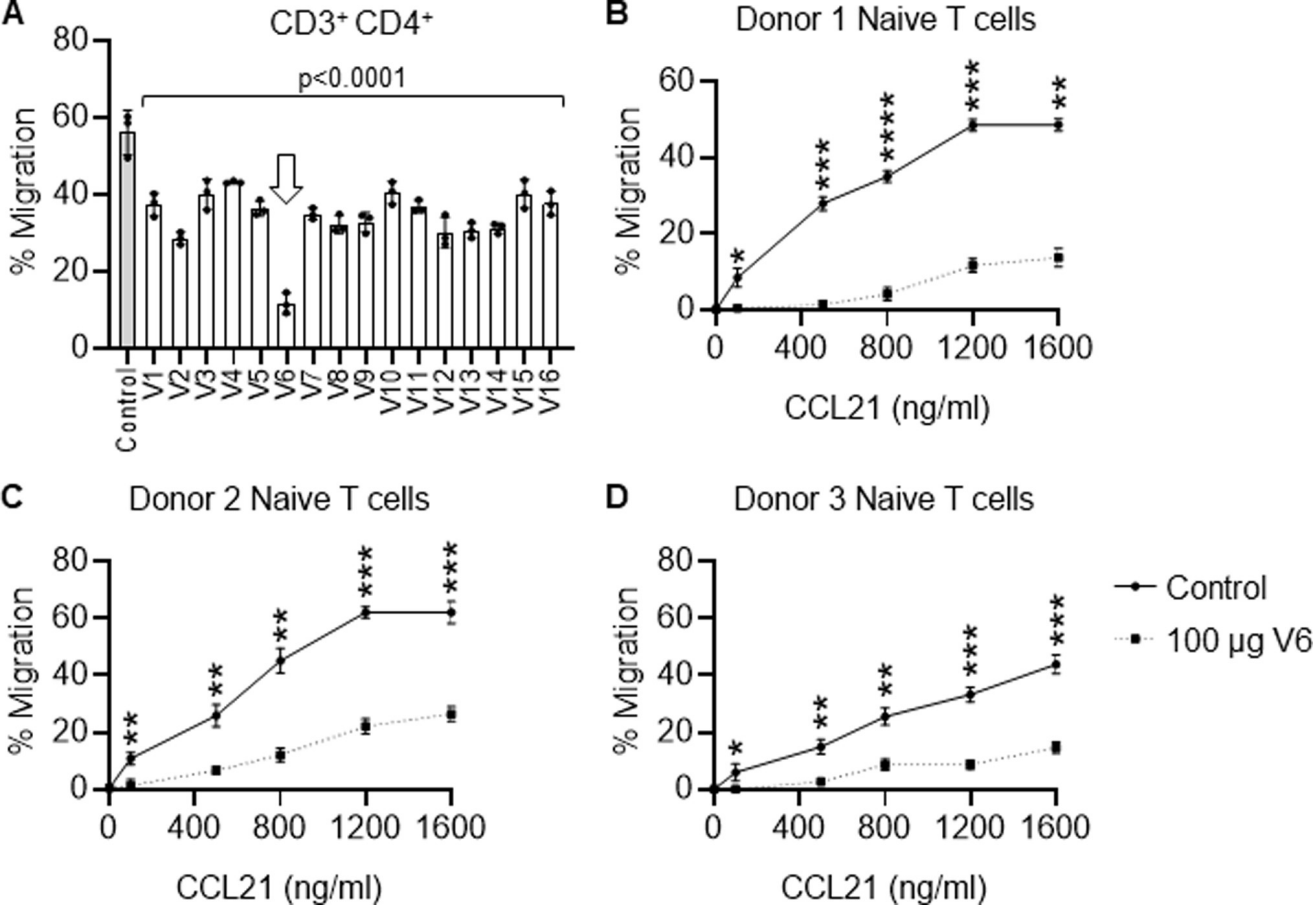
Screening of fully humanized anti-CCL21 monoclonal antibody clones derived from clone C8. **(A)** Inhibition of migration in transwell chemotaxis assays of helper T cells (CD3^+^/CD4^+^) towards 1200 ng/ml rhCCL21 by the 16 humanized clones, referred to as V1 to V16, used at 100 μg/ml. All versions of humanized clones tested inhibited T cell migration towards CCL21 to some degree. However, V6 was the most potent at inhibiting T cell migration towards CCL21 (arrow). Data are the mean ± SD of triplicate wells. One-way ANOVA with post-hoc Tukey’s multiple comparison tests were performed for statistical analysis. All groups had a p<0.0001 when compared to control. **(B-D)** Confirmation of V6 inhibition of CD3^+^/CD4^+^/CD8^-^/CCR7^+^/CD45RA^+^/CD27^+^ Naïve Th-cell chemotaxis towards 100, 500, 800, 1200, and 1600 ng/ml rhCCL21 using CD3^+^ peripheral blood naïve Th-cells from 3 normal human donors. Data are the mean ± SD of triplicate wells.

Sixteen humanized clones (V1 to V16) were generated from C8 and tested for inhibition of chemotaxis of helper T-cells by CCL21. While almost all clones decreased CD3^+^/CD4^+^ Th-cell chemotaxis, interestingly only clone V6 was the most effective humanized clone at blocking helper T-cell migration towards CCL21 (Figs 6 and S9 and S10). This indicates that not all variations during humanization were equal, and most harmed the activity of C8 in blocking T-cell migration towards CCL21. There could be at least four reasons for this; First, the structure of the human constant regions could have oriented the other humanized antibodies away from CCR7, in an opposite direction as C8. Second, the murine constant regions could have had distinct glycosylation patterns compared to the humanized clones which may be important for interference with CCR7 interaction. Third, aggregation of C8 could have been important for its interference between CCL21 and CCR7, and enhanced solubility was selected for in the humanized clones. The humanized clones were all highly soluble, (88.7–99.3%, with V6 having 90.3% existing as a monomer in solution). This is advantageous for clinical antibody production but may have interfered with function. Fourth, the humanized clones except for V6 could have had deleterious motifs for deamidation and isomerization. Interestingly, there is only one amino acid change between V6 (Thr) and V7 (Ser) in the VL region, but this site is facing externally and could potentially block interaction with CCR7 due to the steric hindrance of the added methyl group on Thr. Thus, small changes in monoclonal antibody sequences can generate large differences in function.

Nonetheless, these data demonstrate that the murine-derived monoclonal antibody C8 could be humanized without loss of activity, which would improve its capability as a therapeutic agent.

We previously found that CCL21 expression was consistently induced in the endothelium of T-cell infiltrative autoimmune diseases of the skin, such as psoriasis [15]. Most lymphocytes present in those autoimmune skin samples were CCR7^+^ [15]. Because CCL21 is a potent and specific naïve T-cell and dendritic cell chemokine, these data implied that CCL21’s endothelial induction mediated the aberrant T-cell infiltration of the skin in those diseases. In this study we demonstrated that the CCL21-mediated migration of multiple types of naïve helper T-cells was fully inhibited by the C8 monoclonal antibody and its humanized counterpart V6. In general, as naïve CD4^+^ T-cells mature to antigen-responding cells, they lose expression of CCR7, CD45RA and CD27 [20] and the cell’s ability to migrate towards CCL21 decreases. This is not surprising since CCR7 is the main chemokine receptor for CCL21. Any residual chemotactic activity towards CCL21 after loss of CCR7 is due to the minor receptor for CCL21, CCRL1 [24].

The expression of CCL21 in the inflamed intestinal endothelium raises two important clinically relevant points. First, the monoclonal antibody C8 recognized CCL21 venule endothelial expression in active IBD. Thus, C8 could serve as a diagnostic tool for early activation of IBD, before major tissue destruction or serve as a prognostic tool for severity of IBD. While defining C8’s exact diagnostic or prognostic utility will require further clinical investigation, such a tool would be useful to clinicians, since IBD symptoms often do not mirror tissue biopsy histology. It can be problematic for the clinician caring for IBD patients to differentiate the etiology of intestinal symptoms. Immunohistology using C8 on a bowel biopsy could provide evidence whether or not the symptoms originate from a presentation or relapse of IBD.

The second clinically relevant point is the possibility that CCL21 expression in IBD could mediate the migration of naïve T-cells to the submucosa for local lymphoid auto-antigen presentation. Indeed, CCL21 also promotes migration of naïve dendritic cells as well, which could mature to locally present antigen to the incoming Th-cells [12, 13]. Thus, expression of CCL21 by submucosal venule endothelium could be a key target for disruption of the biology of IBD [5, 7, 8, 16–18]. The humanized monoclonal V6 antibody could serve as a biologic treatment to prevent the migration of T-cells and dendritic cells that ultimately would lead to mucosal destruction [16–18].

There is still a high fraction of IBD patients that suffer multiple intermittent relapses despite high compliance to therapy [16–18]. In addition, there is a significant cohort of treatment resistant IBD for whom new options for effective therapy are sorely needed. Thus, new therapies for high-risk IBD would be beneficial.

We also explored whether a monoclonal antibody could be generated that blocked CCL21-mediated T-cell migration. This would suggest that such an avenue for therapy of IBD could be effective [16–18]. In this study we discovered one monoclonal antibody that blocked the migration of naïve T-cells towards CCL21 and that identified expression of CCL21 on the endothelium of inflamed gut venules. We recognize that development of this biological therapeutic agent will require more extensive pre-clinical study. This made more complex because murine CCL21 is fairly divergent from human CCL21, and C8 does not bind well with murine CCL21 (Fig 1). This was an advantage when generating the murine monoclonal against human CCL21, because the human CCL21 could generate an immune response in the mouse. However, it also means that pre-clinical efficacy testing may require a primate model of IBD.

The timing of therapy with the humanized V6 might be crucial for this agent to be effective. It is possible that this agent might decrease relapse of IBD after remission, but might not treat active disease, since by that time it may be too late to prevent recruitment of naïve T-cells for antigen presentation. Such T-cells would already be activated. Thus, we would hypothesize that CCL21 is crucial for recruitment of naïve T-cells and dendritic cells to sites of mucosal antigen presentation in IBD. This implies it might be better therapy for prevention of relapse than for treatment of active disease. One could also envision other gut inflammatory diseases such as graft versus host disease (GVHD) to be amenable to therapy with CCL21 blockade.

The data here combined with previous reports provides evidence that autoimmune disease of the gut may be distinct from other autoimmune diseases such as rheumatoid arthritis [3–5, 7, 8]. Gut autoimmune disease may result more from local auto-antigen presentation given that the gut Payer’s patches are secondary lymphoid organs. Draining lymph nodes are less likely to be the site of activation of naïve lymphocytes in gut autoimmune disease compared to other target organ autoimmune disease [7, 8, 16–19]. Thus, local expression of venule endothelium CCL21 might be crucial for activation of naive lymphocytes in IBD [17–20]. One could postulate a model for this; aberrant microbiome stimulation of local tissue inflammatory cytokines could activate abnormal CCL21 endothelial expression, which arrests rolling naïve Th-cells, and induces their diapedesis, where they can be presented with auto-antigen [9, 11–14, 19]. The basic, positive-charged carboxy terminus of CCL21 interacts with the negative-charged endothelial heparinoids and hyaluronic acids, maintaining CCL21 in position, and presenting its amino terminus for venule lymphocyte CCR7 binding [11–14, 21]. Blocking naïve T-cell transmigration from the blood to the gut submucosa may lead to a decrease in the pathologic damage of these tissues by locally activated T-cells. This study implies that one method of accomplishing this would be by blocking CCL21 endothelial function with a monoclonal antibody, which may be an important new therapeutic strategy in IBD.

## Conclusion

Using a novel screening method, we identified a murine monoclonal antibody against the human endothelial chemokine CCL21 that blocked naïve and partially activated T-cell chemotaxis towards CCL21. This monoclonal antibody identified expression of CCL21 in active inflammatory diseases of the bowel. However, there was no expression in normal bowel, thus it may be a diagnostic marker for active IBD. We generated multiple distinct humanized clones using the hypervariable region of the C8 murine monoclonal antibody, and one of these, V6 was still effective at blocking T-cell chemotaxis towards CCL21. Thus, this humanized monoclonal antibody against CCL21 may be an effective agent for diagnosis of actively inflamed bowel and may perhaps be a therapeutic agent to prevent relapse of IBD.

## Supporting information

S1 Fig Screening of monoclonal antibody clones for binding to the amino terminus of human CCL21. **A)** Uncropped western blots indicating the murine anti-human CCL21 antibody clones that recognized human CCL21 protein as a single band. **B)** Uncropped slot blot analysis of the monoclonal antibody clones recognizing human and not murine CCL21, since murine CCL21 differs significantly from human CCL21 in the amino terminus, the CCR7-interacting region. **C)** Uncropped slot blot analysis of peptides from the amino terminus of human CCL21 competing off the binding of the clone #8 (termed 3B4-1B2 here) to human CCL21. **D)** Uncropped western blots demonstrating that clone #8 did not recognize the related chemokine CCL19.
(JPG)

S2 Fig Raw data from screening of monoclonal antibody clones 7–15 that passed the screening above for specific binding to the amino terminus of human CCL21 for inhibiting normal human Th-cell chemotaxis towards CCL21. (PDF)

S3 Fig Raw data from screening of monoclonal antibody clones 16–24 that passed the screening above for specific binding to the amino terminus of human CCL21 for inhibiting normal human Th-cell chemotaxis towards CCL21. (PDF)

S4 Fig Raw data from screening of monoclonal antibody clones 25–33 that passed the screening above for specific binding to the amino terminus of human CCL21 for inhibiting normal human Th-cell chemotaxis towards CCL21. (PDF)

S5 Fig Raw data from screening of monoclonal antibody clones 34–39 that passed the screening above for specific binding to the amino terminus of human CCL21 for inhibiting normal human Th-cell chemotaxis towards CCL21. (PDF)

S6 Fig Raw data from measuring the inhibition of three normal human donors’ Th-cell chemotaxis towards CCL21 by clone #8. (PDF)

S7 Fig Raw data from the inhibition of chemotaxis of normal human Th-cell naïve to mature subsets towards CCL21 by C8. (PDF)

S8 Fig Uncropped photomicrographs of immunohistologic analysis of C8 binding to inflammatory bowel biopsy specimens. (PDF)

S9 Fig Raw data from the inhibition of normal human Th-cell chemotaxis towards CCL21 by the 16 different humanized C8 clones. (PDF)

S10 Fig Raw data from the inhibition of chemotaxis of Th-cells from three normal human donors by humanized clone V6. (PDF)
