# Supplementary figures and images for: A humanized monoclonal antibody against the endothelial chemokine CCL21 for the diagnosis and treatment of inflammatory bowel disease
Source: PLoS One. 2021 Jul 1;16(7):e0252805. doi: 10.1371/journal.pone.0252805 (PMC8248966; doi:10.1371/journal.pone.0252805)

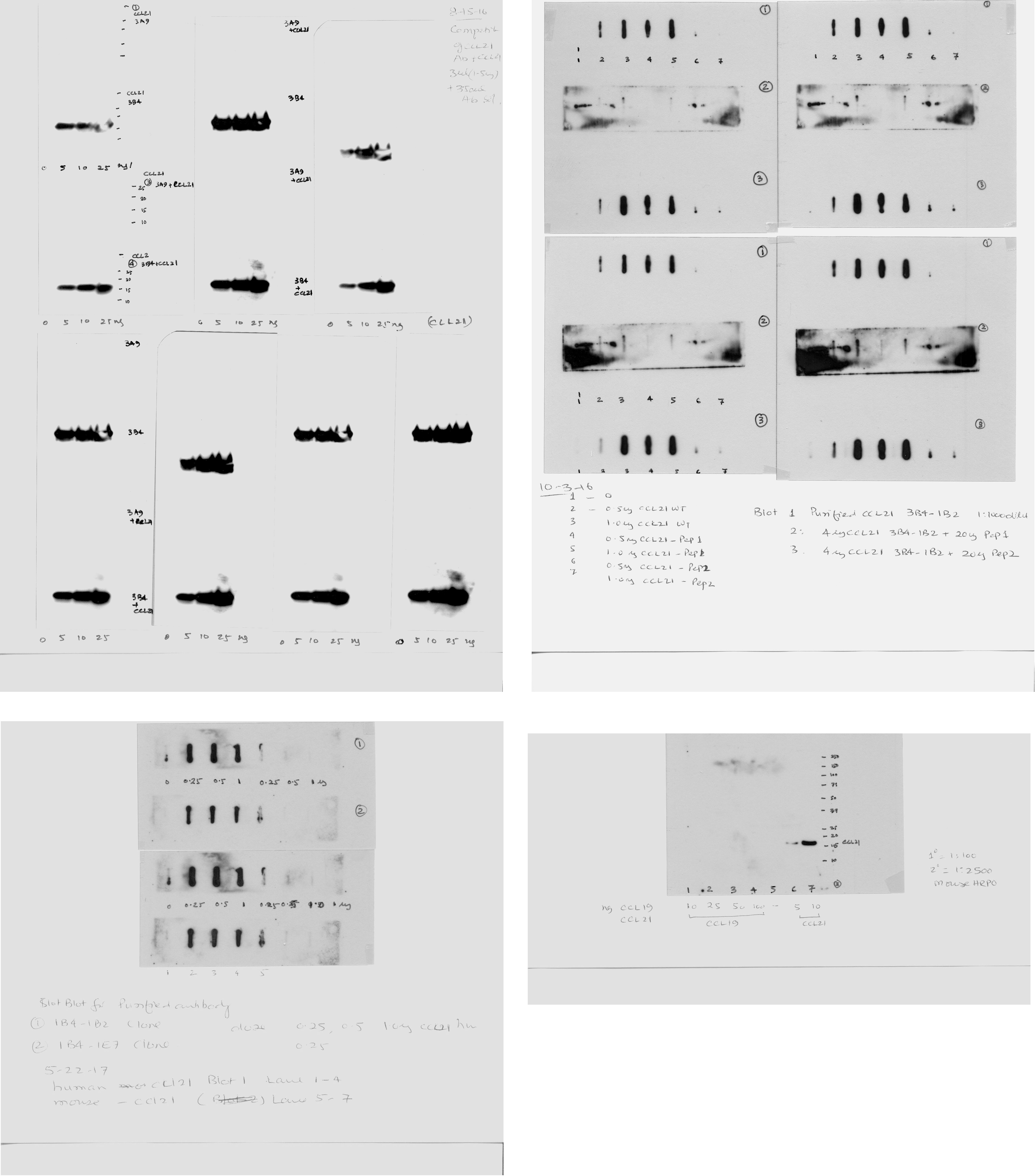

Supplement: S1 Fig — A) Uncropped western blots indicating the murine anti-human CCL21 antibody clones that recognized human CCL21 protein as a single band. B) Uncropped slot blot analysis of the monoclonal antibody clones recognizing human and not murine CCL21, since murine CCL21 differs significantly from human CCL21 in the amino terminus, the CCR7-interacting region. C) Uncropped slot blot analysis of peptides from the amino terminus of human CCL21 competing off the binding of the clone #8 (termed 3B4-1B2 here) to human CCL21. D) Uncropped western blots demonstrating that clone #8 did not recognize the related chemokine CCL19. (JPG) [file pone.0252805.s001.jpg]

IBD biopsies stained for CCL21 using C8

S19-8111 (C1)

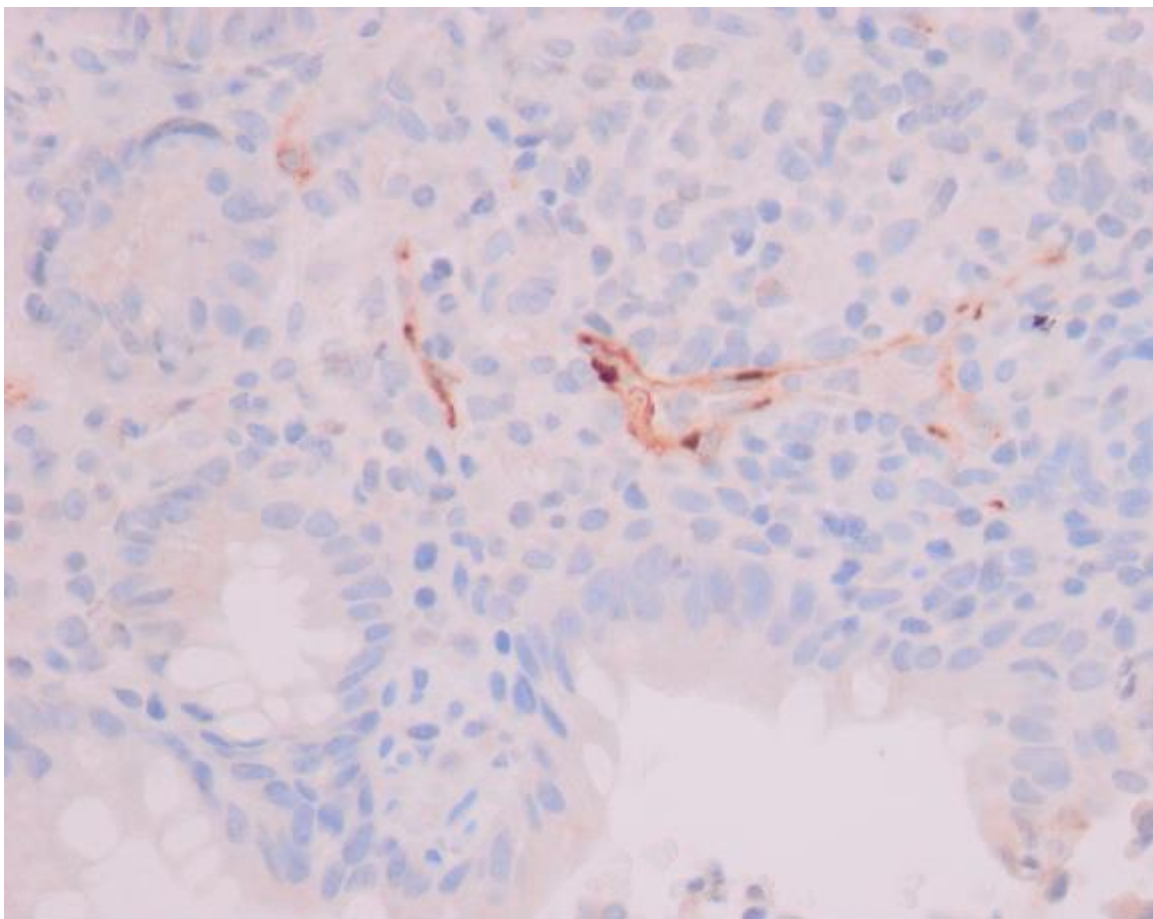

S19-8111 (D1)

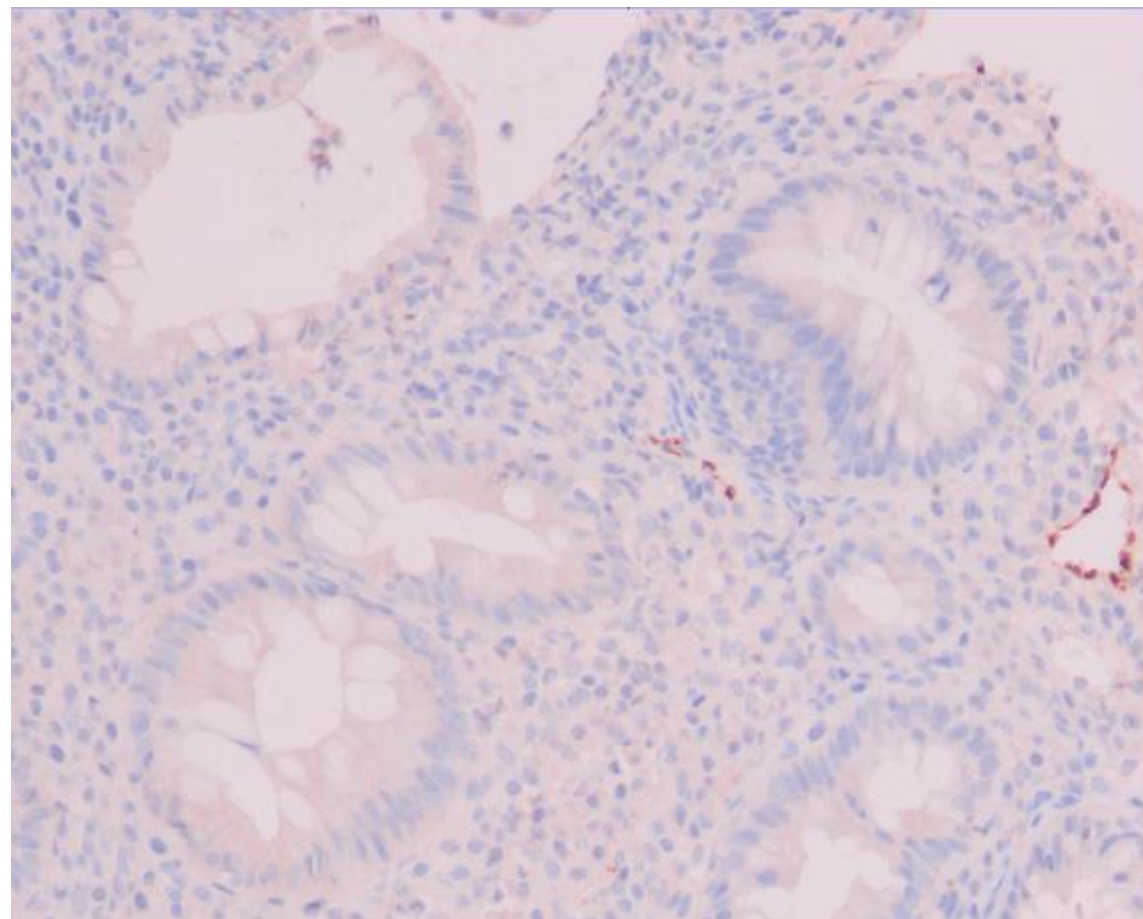

S19-14718 (A1)

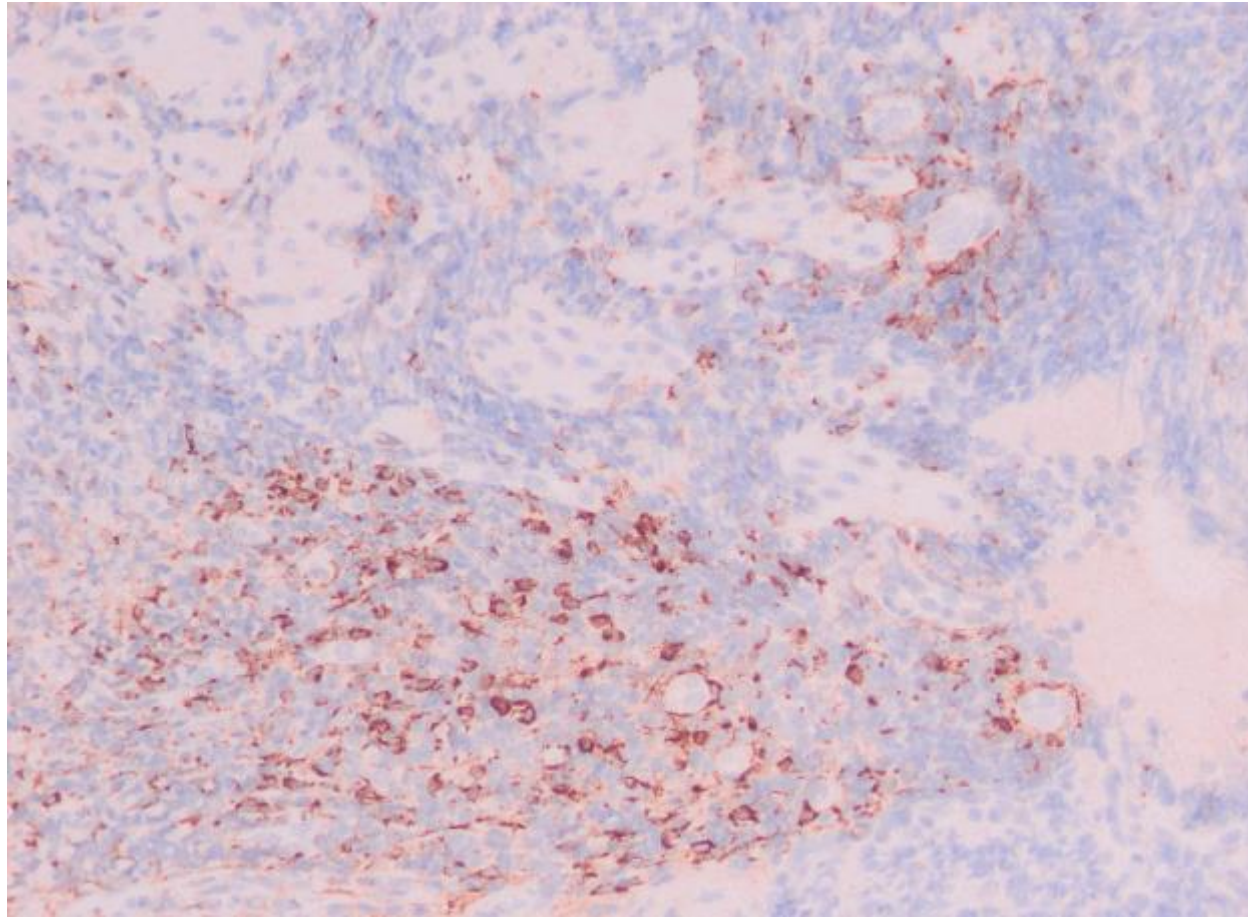

S19-14718 (C1)

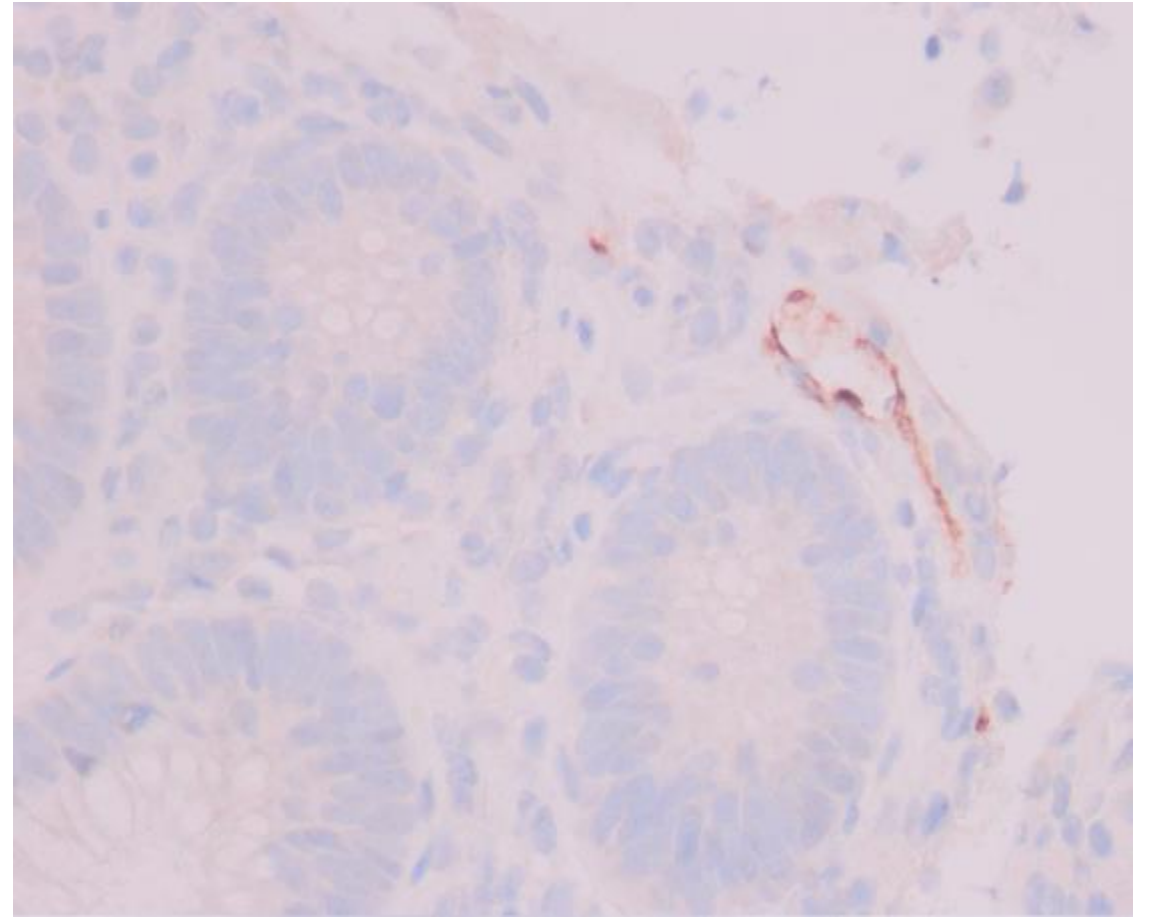

S19-14718 (B1)

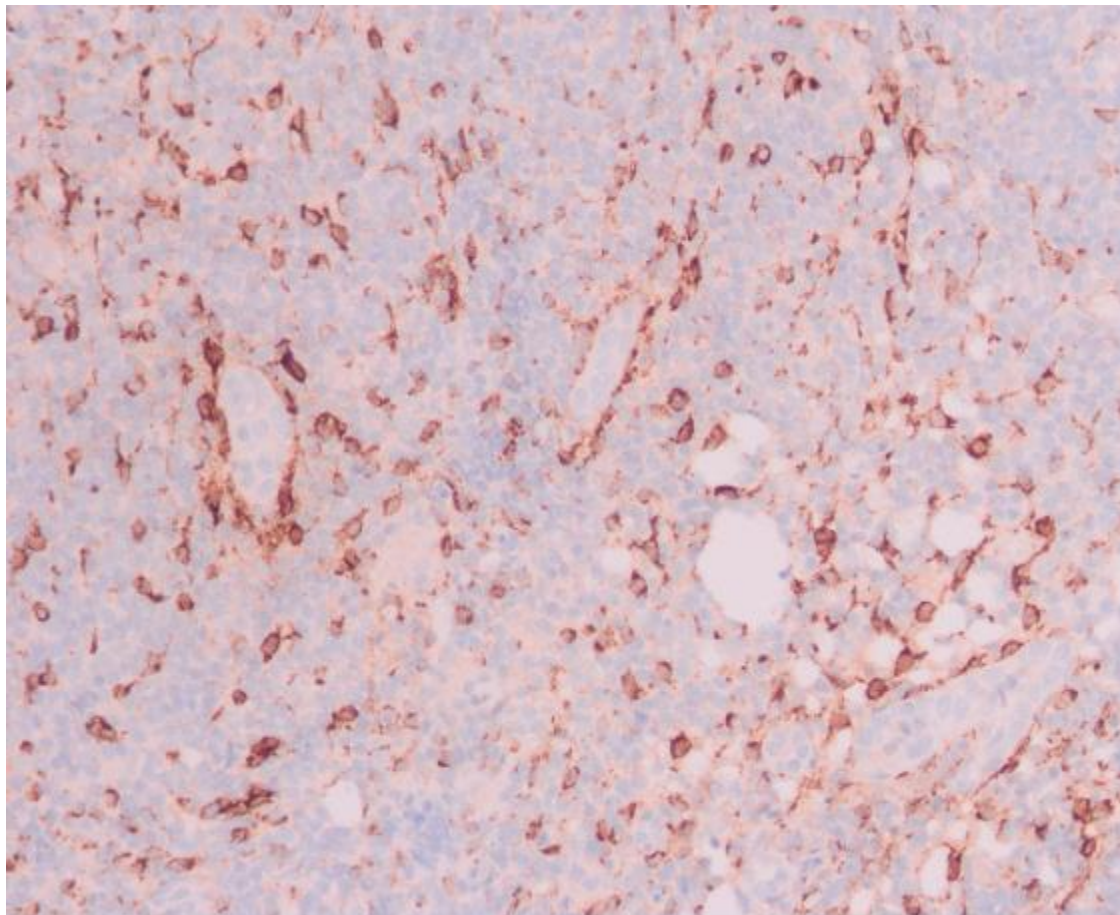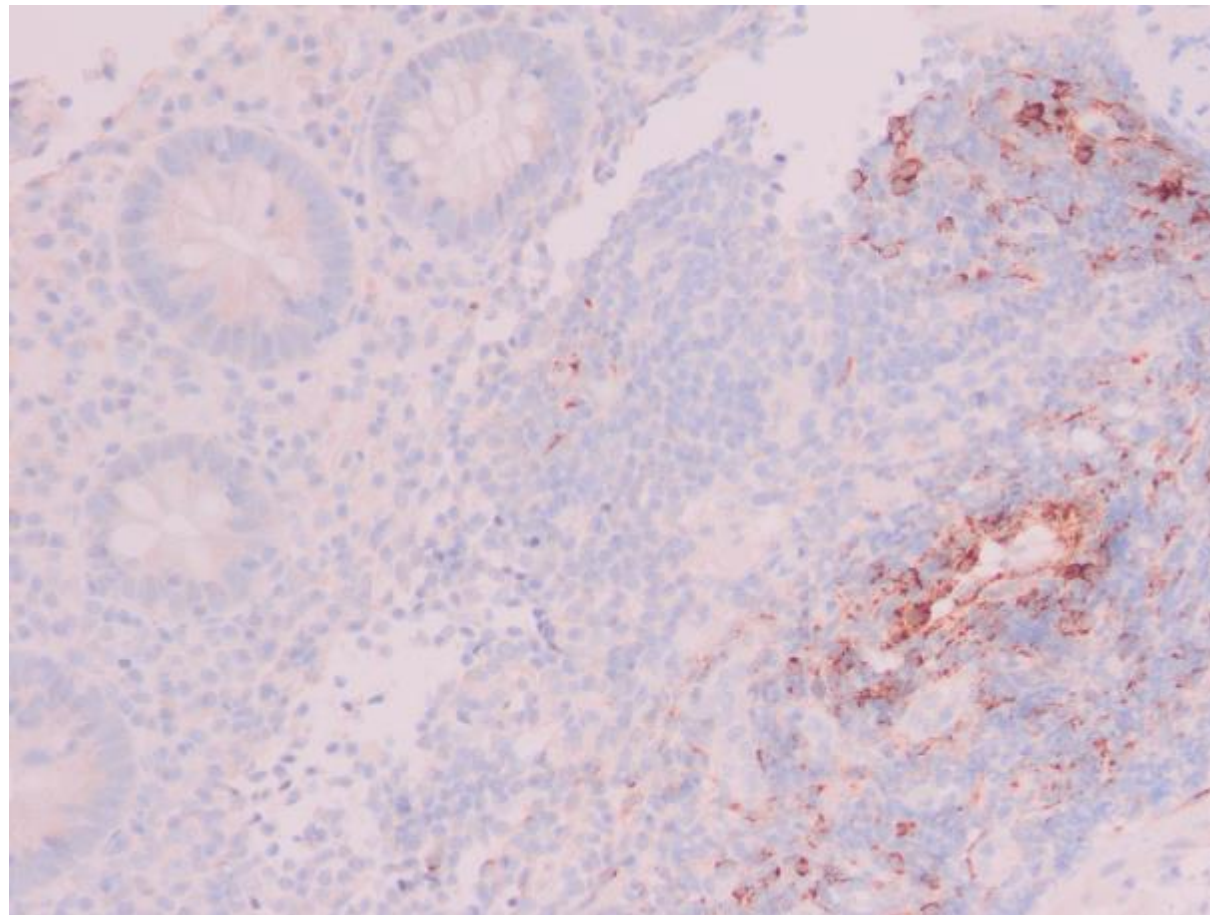

S19-14017 (A1)

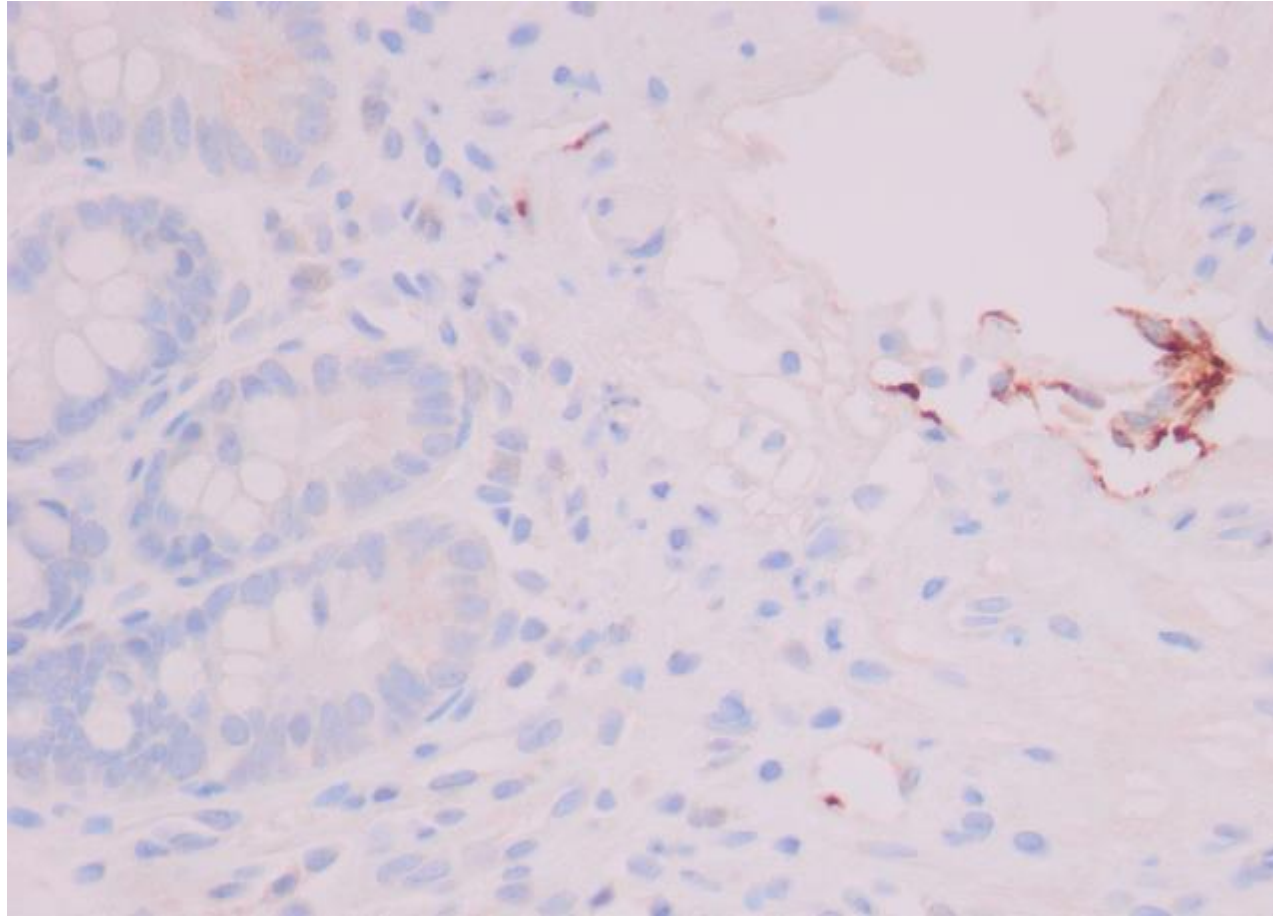

S19-16224 (C1)

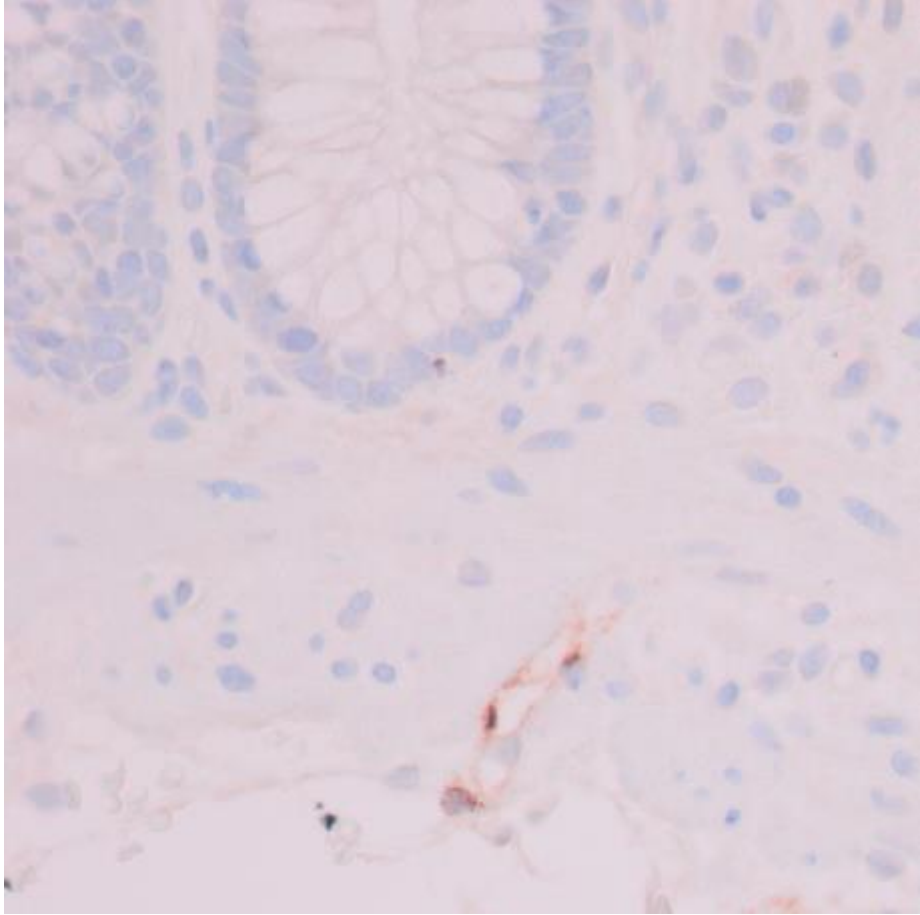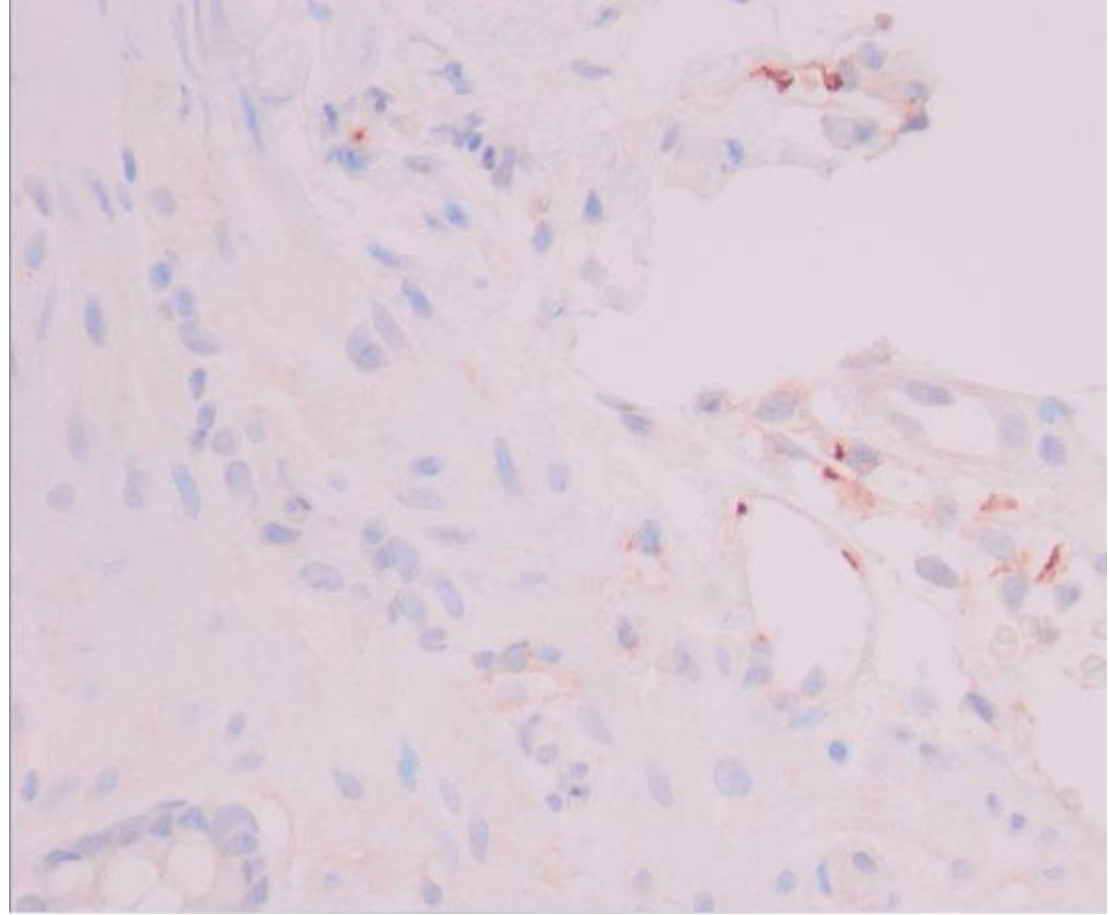

S19-16224 (A1)

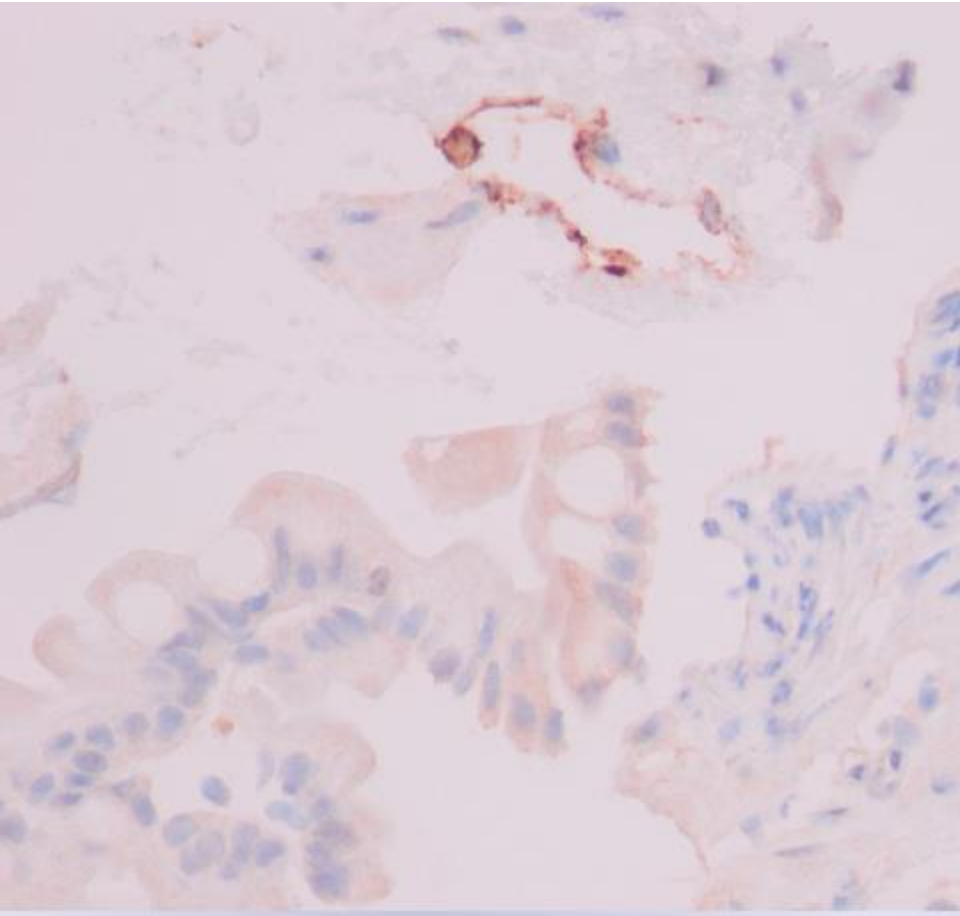

S19-16224 (B1)

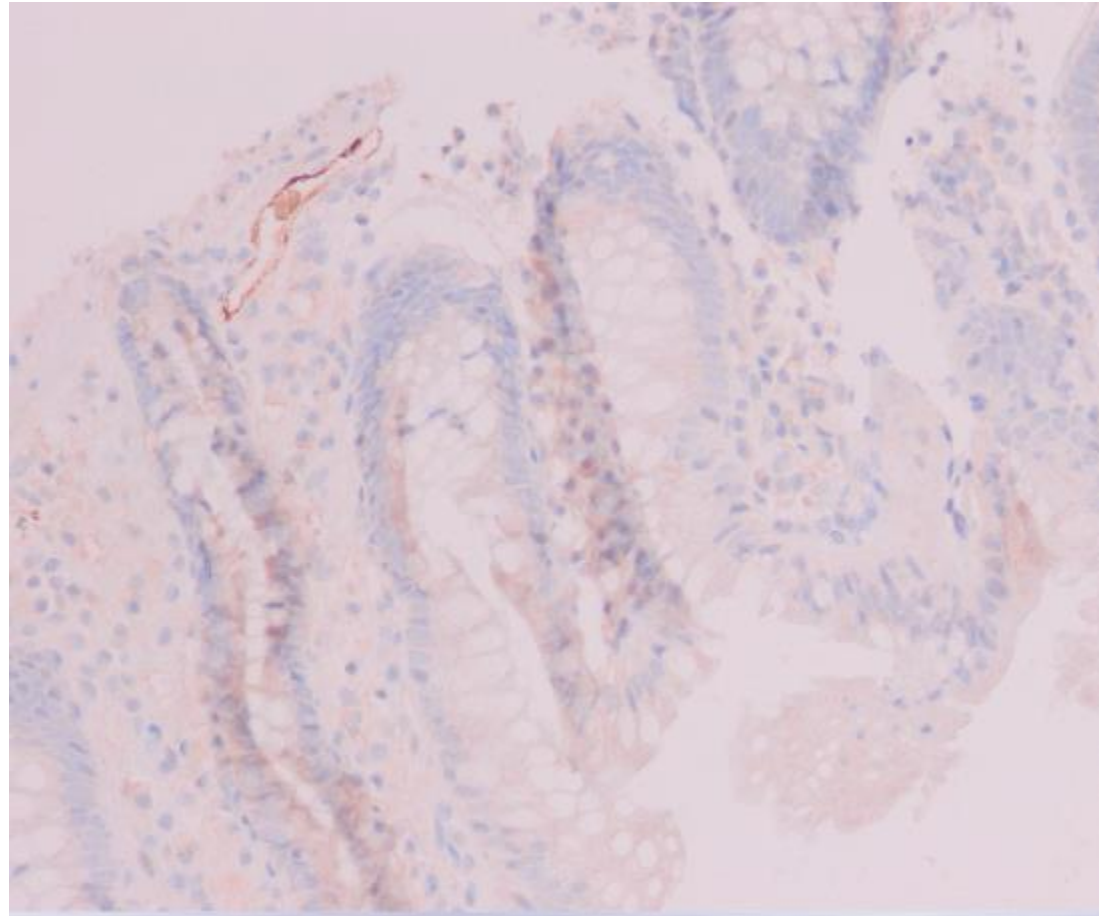

TS19-06556-6A

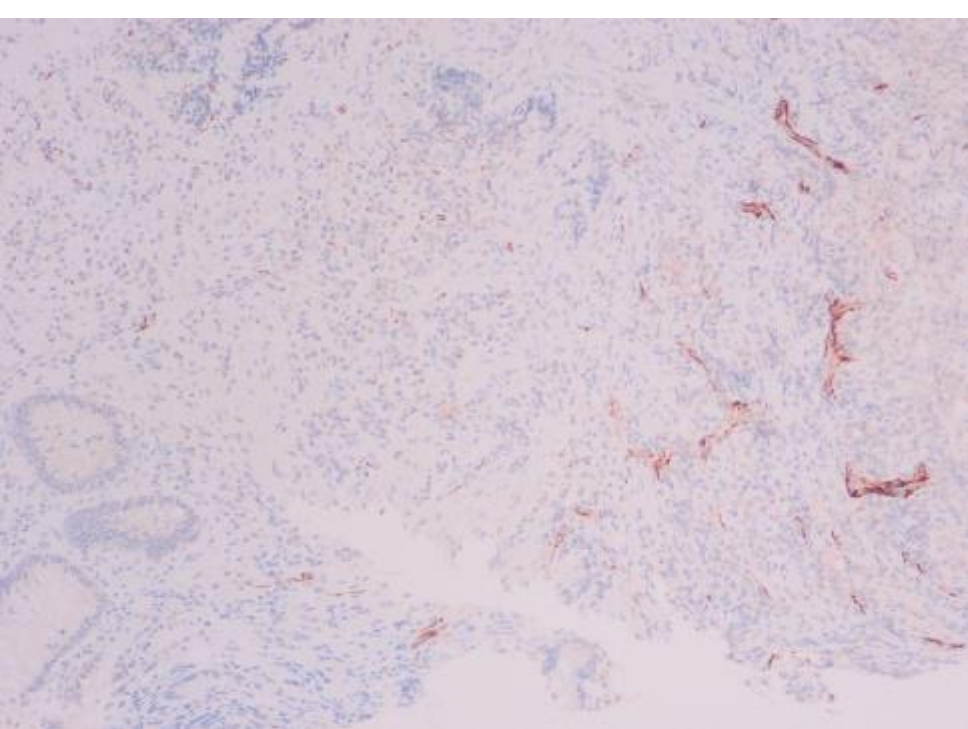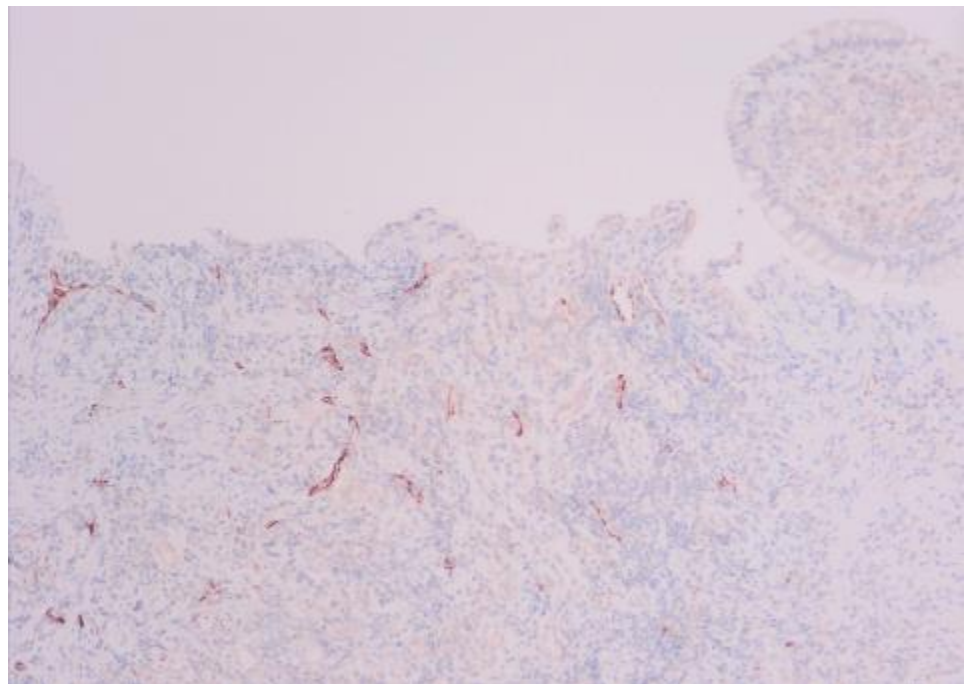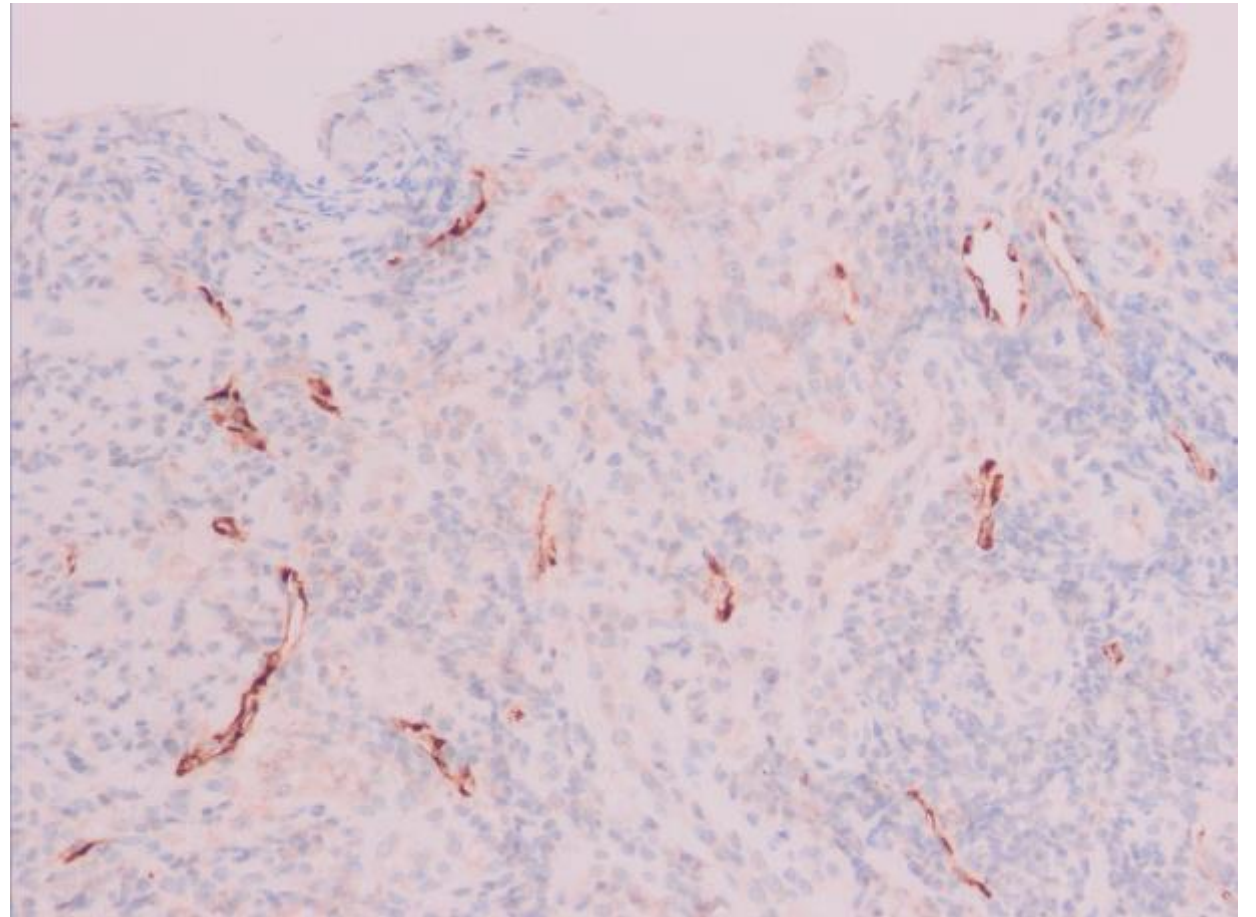

Supplement: S8 Fig — (PDF) [file pone.0252805.s008.pdf]
