# Supplementary material for: A humanized monoclonal antibody against the endothelial chemokine CCL21 for the diagnosis and treatment of inflammatory bowel disease
Source: PLoS One. 2021 Jul 1;16(7):e0252805. doi: 10.1371/journal.pone.0252805 (PMC8248966; doi:10.1371/journal.pone.0252805)
Supplement: S2 Fig — (PDF) [file pone.0252805.s002.pdf]

% Mig CCL21	Well #1	Well #2	Well #3	Ave.
CD3	14.7	16.7	17.6	16.3
CD4	28.4	32.1	33.7	31.4
CD8	6.9	8.7	9.5	8.4
Naïve	33.8	38.0	39.8	37.2
Naïve CD27+	33.8	38.0	39.8	37.2
Group A	27.6	31.3	32.9	30.6
Group A CD27+	27.3	31.0	32.6	30.3
Group B&C	7.2	8.6	9.2	8.3
B&C CD27+	7.0	8.3	8.9	8.1
B&C CD27-	7.5	9.2	10.0	8.9

% Mig #7	Well #1	Well #2	Well #3	Ave.
CD3	15.9	12.3	14.0	14.0
CD4	30.1	23.9	26.8	26.9
CD8	5.2	2.6	3.8	3.9
Naïve	34.4	27.5	30.8	30.9
Naïve CD27+	34.4	27.5	30.8	30.9
Group A	32.7	25.7	29.0	29.1
Group A CD27+	31.9	25.1	28.3	28.5
Group B&C	9.0	6.4	7.7	7.7
B&C CD27+	8.2	5.8	6.9	7.0
B&C CD27-	12.5	8.7	10.5	10.6

% Mig #8	Well #1	Well #2	Well #3	Ave.
CD3	0.3	1.3	1.5	1.1
CD4	0.1	1.1	1.2	0.8
CD8	0.0	0.0	0.2	0.1
Naïve	0.0	0.2	0.3	0.2
Naïve CD27+	0.0	0.2	0.3	0.2
Group A	1.2	2.9	3.2	2.5
Group A CD27+	1.1	2.7	2.9	2.2
Group B&C	0.8	2.5	2.8	2.1
B&C CD27+	0.2	1.6	1.8	1.2
B&C CD27-	3.4	6.8	7.3	5.8

% Mig #9	Well #1	Well #2	Well #3	Ave.
CD3	9.0	7.8	5.6	7.5
CD4	16.9	14.9	11.3	14.4
CD8	2.2	1.1	0.0	1.1
Naïve	18.3	16.2	12.5	15.7
Naïve CD27+	18.3	16.2	12.5	15.7
Group A	20.6	18.1	13.5	17.4
Group A CD27+	19.9	17.5	13.1	16.9
Group B&C	6.8	5.7	3.5	5.3
B&C CD27+	6.0	5.0	3.1	4.7
B&C CD27-	10.4	8.6	5.2	8.1

% Mig #10	Well #1	Well #2	Well #3	Ave.
CD3	16.9	15.2	18.0	16.7
CD4	31.5	28.5	33.3	31.1
CD8	6.1	4.8	6.9	6.0
Naïve	35.7	32.4	37.7	35.3
Naïve CD27+	35.7	32.4	37.7	35.2
Group A	35.4	32.0	37.5	35.0
Group A CD27+	34.6	31.2	36.6	34.1
Group B&C	9.2	8.0	10.0	9.0
B&C CD27+	8.6	7.4	9.2	8.4
B&C CD27-	11.8	10.1	12.8	11.6

% Mig #11	Well #1	Well #2	Well #3	Ave.
CD3	18.0	19.7	18.3	18.7
CD4	34.3	37.2	34.7	35.4
CD8	6.2	7.4	6.4	6.7
Naïve	39.0	42.2	39.5	40.2
Naïve CD27+	38.9	42.1	39.4	40.2
Group A	37.4	40.6	37.9	38.6
Group A CD27+	36.5	39.7	37.0	37.8
Group B&C	10.6	11.8	10.8	11.1
B&C CD27+	10.1	11.2	10.3	10.5
B&C CD27-	13.1	14.8	13.4	13.8

% Mig #12	Well #1	Well #2	Well #3	Ave.
CD3	21.5	23.7	19.0	21.4
CD4	40.7	44.5	36.2	40.5
CD8	8.3	9.8	6.6	8.2
Naïve	47.3	51.7	42.2	47.1
Naïve CD27+	47.3	51.7	42.3	47.1
Group A	41.3	45.3	36.6	41.1
Group A CD27+	40.7	44.7	36.1	40.5
Group B&C	12.4	13.9	10.6	12.3
B&C CD27+	11.3	12.7	9.7	11.2
B&C CD27-	17.1	19.3	14.5	17.0

% Mig #13	Well #1	Well #2	Well #3	Ave.
CD3	19.6	20.7	18.1	19.5
CD4	37.0	38.9	34.3	36.7
CD8	8.6	9.4	7.4	8.5
Naïve	42.1	44.2	39.1	41.8
Naïve CD27+	42.2	44.2	39.1	41.8
Group A	39.4	41.4	36.4	39.1
Group A CD27+	38.7	40.7	35.8	38.4
Group B&C	12.7	13.5	11.5	12.6
B&C CD27+	12.0	12.8	10.9	11.9
B&C CD27-	15.5	16.6	13.9	15.4

% Mig #14	Well #1	Well #2	Well #3	Ave.
CD3	19.6	23.2	20.3	21.0
CD4	37.1	43.5	38.3	39.7
CD8	8.1	10.8	8.6	9.2
Naïve	42.5	49.6	43.8	45.3
Naïve CD27+	42.5	49.6	43.8	45.3
Group A	39.0	45.9	40.3	41.7
Group A CD27+	38.2	45.0	39.5	40.9
Group B&C	12.4	15.1	12.9	13.5
B&C CD27+	11.6	14.2	12.1	12.6
B&C CD27-	15.1	18.8	15.8	16.6

% Mig #15	Well #1	Well #2	Well #3	Ave.
CD3	15.2	14.1	16.6	15.3
CD4	28.0	26.0	30.3	28.1
CD8	6.6	5.7	7.8	6.7
Naïve	31.8	29.6	34.3	31.9
Naïve CD27+	31.8	29.6	34.3	31.9
Group A	29.4	27.2	31.9	29.5
Group A CD27+	29.1	26.9	31.5	29.2
Group B&C	10.5	9.5	11.6	10.5
B&C CD27+	9.7	8.8	10.8	9.8
B&C CD27-	13.7	12.3	15.2	13.7
