# Supplementary material for: A humanized monoclonal antibody against the endothelial chemokine CCL21 for the diagnosis and treatment of inflammatory bowel disease
Source: PLoS One. 2021 Jul 1;16(7):e0252805. doi: 10.1371/journal.pone.0252805 (PMC8248966; doi:10.1371/journal.pone.0252805)
Supplement: S3 Fig — (PDF) [file pone.0252805.s003.pdf]

% Mig CCL21	Well #1	Well #2	Well #3	Ave.
CD3	23.5	23.8	22.1	23.1
CD4	39.4	40.0	37.1	38.8
CD8	21.8	22.2	20.5	21.5
Naïve	42.7	43.4	40.2	42.1
Naïve CD27+	42.7	43.4	40.2	42.1
Group A	38.5	39.1	36.3	38.0
Group A CD27+	38.6	39.2	36.4	38.1
Group B&C	35.7	36.2	33.6	35.2
B&C CD27+	40.7	41.3	38.3	40.1
B&C CD27-	19.9	20.3	18.7	19.6

% Mig #16	Well #1	Well #2	Well #3	Ave.
CD3	22.4	21.4	22.7	22.2
CD4	38.1	36.3	38.6	37.6
CD8	20.4	19.4	20.6	20.1
Naïve	41.0	39.1	41.6	40.6
Naïve CD27+	41.0	39.1	41.6	40.6
Group A	39.6	37.8	40.1	39.2
Group A CD27+	39.7	37.8	40.2	39.2
Group B&C	33.1	31.5	33.5	32.7
B&C CD27+	37.9	36.1	38.4	37.5
B&C CD27-	17.1	16.3	17.3	16.9

% Mig #17	Well #1	Well #2	Well #3	Ave.
CD3	23.6	22.9	24.1	23.5
CD4	40.4	39.3	41.3	40.4
CD8	21.3	20.7	21.8	21.3
Naïve	42.7	41.5	43.7	42.7
Naïve CD27+	42.7	41.5	43.7	42.7
Group A	40.5	39.4	41.4	40.5
Group A CD27+	40.6	39.5	41.5	40.5
Group B&C	40.8	39.6	41.7	40.7
B&C CD27+	47.6	46.2	48.6	47.5
B&C CD27-	19.1	18.5	19.5	19.0

% Mig #18	Well #1	Well #2	Well #3	Ave.
CD3	22.2	21.7	19.8	21.2
CD4	38.0	37.3	34.0	36.4
CD8	20.5	20.1	18.3	19.6
Naïve	39.8	39.0	35.6	38.1
Naïve CD27+	39.8	39.0	35.6	38.1
Group A	39.4	38.6	35.2	37.7
Group A CD27+	39.4	38.7	35.2	37.8
Group B&C	38.0	37.3	33.9	36.4
B&C CD27+	43.8	42.9	39.1	41.9
B&C CD27-	19.7	19.3	17.5	18.8

% Mig #19	Well #1	Well #2	Well #3	Ave.
CD3	23.8	24.6	22.7	23.7
CD4	41.2	42.7	39.3	41.0
CD8	22.5	23.4	21.5	22.5
Naïve	41.3	42.8	39.4	41.2
Naïve CD27+	41.3	42.8	39.4	41.2
Group A	42.6	44.2	40.7	42.5
Group A CD27+	42.7	44.3	40.8	42.6
Group B&C	48.4	50.1	46.1	48.2
B&C CD27+	56.5	58.5	53.8	56.3
B&C CD27-	22.6	23.4	21.5	22.5

% Mig #20	Well #1	Well #2	Well #3	Ave.
CD3	21.0	19.1	19.4	19.8
CD4	35.2	32.1	32.6	33.3
CD8	19.8	18.0	18.3	18.7
Naïve	37.8	34.5	35.0	35.8
Naïve CD27+	37.8	34.5	35.1	35.8
Group A	34.8	31.7	32.3	32.9
Group A CD27+	34.9	31.8	32.4	33.0
Group B&C	34.4	31.3	31.8	32.5
B&C CD27+	40.5	36.8	37.5	38.2
B&C CD27-	14.9	13.4	13.7	14.0

% Mig #21	Well #1	Well #2	Well #3	Ave.
CD3	19.9	17.9	18.5	18.8
CD4	34.7	31.3	32.4	32.8
CD8	18.3	16.5	17.1	17.3
Naïve	37.3	33.7	34.9	35.3
Naïve CD27+	37.3	33.7	34.9	35.3
Group A	35.4	32.0	33.1	33.5
Group A CD27+	35.6	32.1	33.2	33.6
Group B&C	31.3	28.2	29.2	29.6
B&C CD27+	36.3	32.7	33.9	34.3
B&C CD27-	15.2	13.6	14.1	14.3

% Mig #22	Well #1	Well #2	Well #3	Ave.
CD3	22.5	23.5	24.3	23.4
CD4	38.8	40.5	41.8	40.4
CD8	20.9	21.9	22.6	21.8
Naïve	41.0	42.7	44.1	42.6
Naïve CD27+	41.0	42.7	44.1	42.6
Group A	41.1	42.9	44.3	42.8
Group A CD27+	41.2	43.0	44.4	42.9
Group B&C	36.5	38.2	39.4	38.0
B&C CD27+	42.1	43.9	45.4	43.8
B&C CD27-	18.7	19.6	20.2	19.5

% Mig #23	Well #1	Well #2	Well #3	Ave.
CD3	20.0	18.4	18.3	18.9
CD4	34.2	31.5	31.2	32.3
CD8	19.4	17.9	17.7	18.3
Naïve	36.1	33.3	33.0	34.1
Naïve CD27+	36.1	33.3	33.0	34.1
Group A	33.6	31.0	30.7	31.8
Group A CD27+	33.7	31.1	30.8	31.9
Group B&C	35.4	32.5	32.2	33.4
B&C CD27+	41.6	38.3	37.9	39.3
B&C CD27-	15.3	14.1	13.9	14.4

% Mig #24	Well #1	Well #2	Well #3	Ave.
CD3	23.3	22.4	23.9	23.2
CD4	40.8	39.3	41.9	40.7
CD8	20.8	20.0	21.3	20.7
Naïve	44.3	42.6	45.4	44.1
Naïve CD27+	44.3	42.6	45.4	44.1
Group A	42.3	40.7	43.4	42.1
Group A CD27+	42.3	40.7	43.4	42.1
Group B&C	34.9	33.6	35.8	34.8
B&C CD27+	40.3	38.7	41.3	40.1
B&C CD27-	18.2	17.5	18.7	18.2
