# Supplementary material for: A humanized monoclonal antibody against the endothelial chemokine CCL21 for the diagnosis and treatment of inflammatory bowel disease
Source: PLoS One. 2021 Jul 1;16(7):e0252805. doi: 10.1371/journal.pone.0252805 (PMC8248966; doi:10.1371/journal.pone.0252805)
Supplement: S4 Fig — (PDF) [file pone.0252805.s004.pdf]

| % Mig CCL21 | Well #1 | Well #2 | Well #3 | Ave. |
|---|---|---|---|---|
| CD3 | 23.5 | 23.8 | 22.1 | 23.1 |
| CD4 | 39.4 | 40.0 | 37.1 | 38.8 |
| CD8 | 21.8 | 22.2 | 20.5 | 21.5 |
| Naïve | 42.7 | 43.4 | 40.2 | 42.1 |
| Naïve CD27+ | 42.7 | 43.4 | 40.2 | 42.1 |
| Group A | 38.5 | 39.1 | 36.3 | 38.0 |
| Group A CD27+ | 38.6 | 39.2 | 36.4 | 38.1 |
| Group B&C | 35.7 | 36.2 | 33.6 | 35.2 |
| B&C CD27+ | 40.7 | 41.3 | 38.3 | 40.1 |
| B&C CD27- | 19.9 | 20.3 | 18.7 | 19.6 |

| % Mig #25 | Well #1 | Well #2 | Well #3 | Ave. |
|---|---|---|---|---|
| CD3 | 26.0 | 26.3 | 24.8 | 25.7 |
| CD4 | 44.0 | 44.5 | 42.0 | 43.5 |
| CD8 | 24.6 | 24.9 | 23.5 | 24.3 |
| Naïve | 48.1 | 48.6 | 45.9 | 47.5 |
| Naïve CD27+ | 48.1 | 48.6 | 45.9 | 47.5 |
| Group A | 45.4 | 45.9 | 43.3 | 44.9 |
| Group A CD27+ | 45.4 | 46.0 | 43.4 | 44.9 |
| Group B&C | 37.1 | 37.5 | 35.3 | 36.6 |
| B&C CD27+ | 43.3 | 43.8 | 41.3 | 42.8 |
| B&C CD27- | 16.7 | 16.9 | 15.9 | 16.5 |

| % Mig #26 | Well #1 | Well #2 | Well #3 | Ave. |
|---|---|---|---|---|
| CD3 | 27.4 | 25.4 | 28.0 | 26.9 |
| CD4 | 47.5 | 44.0 | 48.5 | 46.7 |
| CD8 | 26.2 | 24.3 | 26.8 | 25.8 |
| Naïve | 51.6 | 47.8 | 52.7 | 50.7 |
| Naïve CD27+ | 51.6 | 47.8 | 52.7 | 50.7 |
| Group A | 50.3 | 46.6 | 51.4 | 49.4 |
| Group A CD27+ | 50.4 | 46.7 | 51.5 | 49.5 |
| Group B&C | 39.0 | 36.1 | 39.9 | 38.3 |
| B&C CD27+ | 45.3 | 41.9 | 46.3 | 44.5 |
| B&C CD27- | 18.2 | 16.8 | 18.6 | 17.9 |

| % Mig #27 | Well #1 | Well #2 | Well #3 | Ave. |
|---|---|---|---|---|
| CD3 | 22.1 | 22.8 | 24.5 | 23.1 |
| CD4 | 37.7 | 38.9 | 41.7 | 39.4 |
| CD8 | 20.9 | 21.6 | 23.2 | 21.9 |
| Naïve | 40.3 | 41.5 | 44.6 | 42.1 |
| Naïve CD27+ | 40.3 | 41.5 | 44.6 | 42.1 |
| Group A | 39.8 | 41.0 | 44.1 | 41.6 |
| Group A CD27+ | 39.9 | 41.1 | 44.2 | 41.7 |
| Group B&C | 33.5 | 34.6 | 37.2 | 35.1 |
| B&C CD27+ | 39.1 | 40.4 | 43.4 | 40.9 |
| B&C CD27- | 16.0 | 16.5 | 17.8 | 16.8 |

| % Mig #28 | Well #1 | Well #2 | Well #3 | Ave. |
|---|---|---|---|---|
| CD3 | 25.2 | 26.4 | 25.0 | 25.6 |
| CD4 | 43.1 | 45.0 | 42.7 | 43.6 |
| CD8 | 24.4 | 25.5 | 24.2 | 24.7 |
| Naïve | 45.4 | 47.4 | 45.1 | 46.0 |
| Naïve CD27+ | 45.4 | 47.4 | 45.1 | 46.0 |
| Group A | 45.4 | 47.4 | 45.1 | 46.0 |
| Group A CD27+ | 45.6 | 47.6 | 45.2 | 46.1 |
| Group B&C | 40.9 | 42.8 | 40.6 | 41.4 |
| B&C CD27+ | 47.6 | 49.7 | 47.2 | 48.1 |
| B&C CD27- | 20.3 | 21.2 | 20.1 | 20.6 |

| % Mig #29 | Well #1 | Well #2 | Well #3 | Ave. |
|---|---|---|---|---|
| CD3 | 20.7 | 19.9 | 19.2 | 19.9 |
| CD4 | 35.2 | 33.9 | 32.7 | 33.9 |
| CD8 | 19.4 | 18.7 | 18.0 | 18.7 |
| Naïve | 37.1 | 35.7 | 34.5 | 35.8 |
| Naïve CD27+ | 37.1 | 35.7 | 34.5 | 35.8 |
| Group A | 37.8 | 36.4 | 35.2 | 36.5 |
| Group A CD27+ | 37.8 | 36.4 | 35.1 | 36.4 |
| Group B&C | 31.8 | 30.6 | 29.5 | 30.6 |
| B&C CD27+ | 36.8 | 35.4 | 34.2 | 35.5 |
| B&C CD27- | 15.0 | 14.5 | 13.9 | 14.5 |

| % Mig #30 | Well #1 | Well #2 | Well #3 | Ave. |
|---|---|---|---|---|
| CD3 | 23.7 | 23.0 | 24.5 | 23.8 |
| CD4 | 42.8 | 41.5 | 44.3 | 42.8 |
| CD8 | 20.2 | 19.5 | 20.8 | 20.2 |
| Naïve | 46.3 | 44.9 | 47.9 | 46.4 |
| Naïve CD27+ | 46.3 | 44.9 | 47.9 | 46.4 |
| Group A | 47.7 | 46.1 | 49.2 | 47.7 |
| Group A CD27+ | 47.7 | 46.2 | 49.3 | 47.8 |
| Group B&C | 32.9 | 31.9 | 34.1 | 32.9 |
| B&C CD27+ | 37.3 | 36.1 | 38.6 | 37.3 |
| B&C CD27- | 19.5 | 18.9 | 20.2 | 19.5 |

| % Mig #31 | Well #1 | Well #2 | Well #3 | Ave. |
|---|---|---|---|---|
| CD3 | 22.5 | 21.3 | 21.6 | 21.8 |
| CD4 | 41.7 | 39.5 | 40.1 | 40.5 |
| CD8 | 19.8 | 18.7 | 19.0 | 19.2 |
| Naïve | 42.2 | 40.0 | 40.6 | 40.9 |
| Naïve CD27+ | 42.2 | 40.0 | 40.6 | 40.9 |
| Group A | 46.5 | 44.0 | 44.7 | 45.1 |
| Group A CD27+ | 46.5 | 44.1 | 44.8 | 45.1 |
| Group B&C | 44.2 | 41.8 | 42.5 | 42.8 |
| B&C CD27+ | 51.2 | 48.5 | 49.2 | 49.6 |
| B&C CD27- | 23.0 | 21.7 | 22.0 | 22.2 |

| % Mig #32 | Well #1 | Well #2 | Well #3 | Ave. |
|---|---|---|---|---|
| CD3 | 23.6 | 24.0 | 22.8 | 23.4 |
| CD4 | 41.9 | 42.6 | 40.5 | 41.7 |
| CD8 | 21.6 | 22.0 | 20.9 | 21.5 |
| Naïve | 45.3 | 46.1 | 43.8 | 45.0 |
| Naïve CD27+ | 45.3 | 46.1 | 43.8 | 45.0 |
| Group A | 42.8 | 43.6 | 41.4 | 42.6 |
| Group A CD27+ | 42.9 | 43.7 | 41.5 | 42.7 |
| Group B&C | 37.4 | 38.1 | 36.1 | 37.2 |
| B&C CD27+ | 43.9 | 44.7 | 42.4 | 43.7 |
| B&C CD27- | 15.9 | 16.2 | 15.4 | 15.8 |

| % Mig #33 | Well #1 | Well #2 | Well #3 | Ave. |
|---|---|---|---|---|
| CD3 | 23.2 | 24.5 | 21.6 | 23.1 |
| CD4 | 40.1 | 42.3 | 37.3 | 39.9 |
| CD8 | 22.1 | 23.3 | 20.6 | 22.0 |
| Naïve | 42.8 | 45.1 | 39.8 | 42.6 |
| Naïve CD27+ | 42.8 | 45.1 | 39.8 | 42.6 |
| Group A | 43.5 | 45.9 | 40.4 | 43.3 |
| Group A CD27+ | 43.6 | 45.9 | 40.5 | 43.3 |
| Group B&C | 34.0 | 35.9 | 31.6 | 33.8 |
| B&C CD27+ | 39.0 | 41.2 | 36.3 | 38.8 |
| B&C CD27- | 18.3 | 19.4 | 17.0 | 18.2 |
