# Supplementary material for: A humanized monoclonal antibody against the endothelial chemokine CCL21 for the diagnosis and treatment of inflammatory bowel disease
Source: PLoS One. 2021 Jul 1;16(7):e0252805. doi: 10.1371/journal.pone.0252805 (PMC8248966; doi:10.1371/journal.pone.0252805)
Supplement: S5 Fig — (PDF) [file pone.0252805.s005.pdf]

% Mig CCL21	Well #1	Well #2	Well #3	Ave.
CD3	23.5	23.8	22.1	23.1
CD4	39.4	40.0	37.1	38.8
CD8	21.8	22.2	20.5	21.5
Naïve	42.7	43.4	40.2	42.1
Naïve CD27+	42.7	43.4	40.2	42.1
Group A	38.5	39.1	36.3	38.0
Group A CD27+	38.6	39.2	36.4	38.1
Group B&C	35.7	36.2	33.6	35.2
B&C CD27+	40.7	41.3	38.3	40.1
B&C CD27-	19.9	20.3	18.7	19.6

% Mig #34	Well #1	Well #2	Well #3	Ave.
CD3	20.1	19.8	18.5	19.4
CD4	34.8	34.3	32.2	33.8
CD8	18.7	18.4	17.2	18.1
Naïve	37.3	36.7	34.4	36.1
Naïve CD27+	37.3	36.7	34.4	36.1
Group A	36.9	36.4	34.1	35.8
Group A CD27+	37.0	36.4	34.2	35.9
Group B&C	30.2	29.7	27.8	29.3
B&C CD27+	35.3	34.8	32.6	34.2
B&C CD27-	12.8	12.6	11.8	12.4

% Mig #35	Well #1	Well #2	Well #3	Ave.
CD3	19.7	18.5	18.0	18.8
CD4	34.7	32.6	31.8	33.0
CD8	17.9	16.8	16.3	17.0
Naïve	36.5	34.2	33.4	34.7
Naïve CD27+	36.5	34.2	33.4	34.7
Group A	37.3	35.0	34.2	35.5
Group A CD27+	37.4	35.1	34.2	35.6
Group B&C	32.1	30.1	29.3	30.5
B&C CD27+	37.4	35.1	34.2	35.5
B&C CD27-	14.8	13.8	13.4	14.0

% Mig #36	Well #1	Well #2	Well #3	Ave.
CD3	21.8	22.7	23.1	22.5
CD4	40.2	41.8	42.5	41.5
CD8	17.5	18.2	18.5	18.0
Naïve	41.5	43.1	43.9	42.9
Naïve CD27+	41.5	43.1	43.9	42.9
Group A	43.8	45.5	46.3	45.2
Group A CD27+	43.9	45.6	46.5	45.3
Group B&C	39.0	40.6	41.3	40.3
B&C CD27+	45.5	47.3	48.2	47.0
B&C CD27-	18.4	19.2	19.5	19.0

% Mig #37	Well #1	Well #2	Well #3	Ave.
CD3	22.2	21.8	21.3	21.8
CD4	38.9	38.2	37.3	38.2
CD8	20.1	19.7	19.3	19.7
Naïve	40.8	40.1	39.1	40.0
Naïve CD27+	40.8	40.1	39.1	40.0
Group A	40.7	40.0	39.0	39.9
Group A CD27+	40.7	40.0	39.1	40.0
Group B&C	38.1	37.4	36.5	37.3
B&C CD27+	44.6	43.9	42.8	43.8
B&C CD27-	16.6	16.3	15.9	16.3

% Mig #38	Well #1	Well #2	Well #3	Ave.
CD3	19.9	18.8	19.4	19.4
CD4	34.1	32.2	33.3	33.2
CD8	19.1	18.1	18.7	18.6
Naïve	35.6	33.7	34.8	34.7
Naïve CD27+	35.6	33.7	34.8	34.7
Group A	35.8	33.9	35.0	34.9
Group A CD27+	35.9	34.0	35.1	35.0
Group B&C	33.5	31.7	32.7	32.6
B&C CD27+	39.4	37.2	38.4	38.3
B&C CD27-	14.5	13.7	14.1	14.1

% Mig #39	Well #1	Well #2	Well #3	Ave.
CD3	21.3	21.7	22.3	21.8
CD4	36.2	36.9	37.8	37.0
CD8	20.3	20.6	21.1	20.7
Naïve	38.1	38.8	39.8	38.9
Naïve CD27+	38.1	38.8	39.8	38.9
Group A	39.3	40.0	41.0	40.1
Group A CD27+	39.3	40.0	41.0	40.1
Group B&C	32.8	33.3	34.2	33.4
B&C CD27+	38.0	38.7	39.6	38.8
B&C CD27-	16.1	16.4	16.9	16.5
