# Supplementary material for: A humanized monoclonal antibody against the endothelial chemokine CCL21 for the diagnosis and treatment of inflammatory bowel disease
Source: PLoS One. 2021 Jul 1;16(7):e0252805. doi: 10.1371/journal.pone.0252805 (PMC8248966; doi:10.1371/journal.pone.0252805)
Supplement: S6 Fig — (PDF) [file pone.0252805.s006.pdf]

	% Migration	
Donor 1 CD3+ CD4+	Control	Clone #8
Well 1	28	0
Well 2	32	1
Well 3	34	1
Average	31.3	0.7

	% Migration	
Donor 2 CD3+ CD4+	Control	Clone #8
Well 1	47.9	6.4
Well 2	54.9	10.4
Well 3	46.3	3.2
Average	49.7	6.7

	% Migration	
Donor 3 CD3+ CD4+	Control	Clone #8
Well 1	64.8	12.4
Well 2	57.1	17.2
Well 3	60.4	8.3
Average	60.8	12.6
