# Supplementary material for: A humanized monoclonal antibody against the endothelial chemokine CCL21 for the diagnosis and treatment of inflammatory bowel disease
Source: PLoS One. 2021 Jul 1;16(7):e0252805. doi: 10.1371/journal.pone.0252805 (PMC8248966; doi:10.1371/journal.pone.0252805)
Supplement: S7 Fig — (PDF) [file pone.0252805.s007.pdf]

# Cells	Input
CD3	152080
CD4	71325
CD8	21900
Naïve	45862
Naïve CD27+	45816
Group A	14622
Group A CD27+	14285
Group B&C	9558
B&C CD27+	7942
B&C CD27-	1491

CCL21-none	Well #1	Well #2	Well #3
CD3	22300	25456	26802
CD4	20271	22896	24016
CD8	1513	1914	2085
Naïve	15509	17439	18262
Naïve CD27+	15486	17412	18234
Group A	4031	4577	4810
Group A CD27+	3906	4433	4658
Group B&C	683	818	875
B&C CD27+	556	663	708
B&C CD27-	111	138	149

% Mig CCL21	Well #1	Well #2	Well #3	Ave.
CD3	15	17	18	16
CD4	28	32	34	31
CD8	7	9	10	8
Naïve	34	38	40	37
Naïve CD27+	34	38	40	37
Group A	28	31	33	31
Group A CD27+	27	31	33	30
Group B&C	7	9	9	8
B&C CD27+	7	8	9	8
B&C CD27-	7	9	10	9

# 7-none	Well #1	Well #2	Well #3
CD3	24109	18694	21239
CD4	21488	17037	19129
CD8	1137	563	833
Naïve	15787	12632	14115
Naïve CD27+	15764	12614	14095
Group A	4782	3762	4241
Group A CD27+	4560	3590	4046
Group B&C	864	614	732
B&C CD27+	650	462	550
B&C CD27-	187	130	157

St. Dev.	% Mig #7	Well #1	Well #2	Well #3	Ave.	St. Dev.
2	CD3	16	12	14	14	2
3	CD4	30	24	27	27	3
1	CD8	5	3	4	4	1
3	Naïve	34	28	31	31	3
3	Naïve CD27+	34	28	31	31	3
3	Group A	33	26	29	29	3
3	Group A CD27+	32	25	28	28	3
1	Group B&C	9	6	8	8	1
1	B&C CD27+	8	6	7	7	1
1	B&C CD27-	13	9	11	11	2

	p Values to CCL21 alone
CD3	0.1605
CD4	0.1439
CD8	**0.0426**
Naïve	0.1059
Naïve CD27+	0.1059
Group A	0.3535
Group A CD27+	0.3120
Group B&C	0.3440
B&C CD27+	0.2185
B&C CD27-	0.2167

# 8-none	Well #1	Well #2	Well #3
CD3	469	2050	2305
CD4	64	758	870
CD8	0	0	36
Naïve	0	90	132
Naïve CD27+	0	91	133
Group A	179	430	471
Group A CD27+	151	382	419
Group B&C	77	243	270
B&C CD27+	15	126	144
B&C CD27-	50	101	109

9-none
CD3
CD4
CD8
Naïve
Naïve CD27+
Group A
Group A CD27+
Group B&C
B&C CD27+
B&C CD27-

% Mig #8	Well #1	Well #2	Well #3	Ave.	St. Dev.
CD3	0	1	2	1	1
CD4	0	1	1	1	1
CD8	0	0	0	0	0
Naïve	0	0	0	0	0
Naïve CD27+	0	0	0	0	0
Group A	1	3	3	2	1
Group A CD27+	1	3	3	2	1
Group B&C	1	3	3	2	1
B&C CD27+	0	2	2	1	1
B&C CD27-	3	7	7	6	2

% Mig #9
CD3
CD4
CD8
Naïve
Naïve CD27+
Group A
Group A CD27+
Group B&C
B&C CD27+
B&C CD27-

	p Values to CCL21 alone		
CD3	**0.0006**		CD3
CD4	**0.0008**		CD4
CD8	**0.0038**		CD8
Naïve	**0.0010**		Naïve
Naïve CD27+	**0.0010**		Naïve CD27+
Group A	**0.0006**		Group A
Group A CD27+	**0.0006**		Group A CD27+
Group B&C	**0.0002**		Group B&C
B&C CD27+	**0.0001**		B&C CD27+
B&C CD27-	**0.0133**		B&C CD27-

Well #1	Well #2	Well #3			# 10-none	Well #1	Well #2
13750	11912	8520			CD3	25769	23105
12085	10663	8038			CD4	22487	20328
482	243	-197			CD8	1343	1058
8377	7443	5718			Naïve	16372	14853
8378	7444	5721			Naïve CD27+	16347	14831
3015	2650	1978			Group A	5183	4674
2849	2505	1870			Group A CD27+	4942	4458
652	541	335			Group B&C	881	763
480	397	245			B&C CD27+	680	590
155	128	78			B&C CD27-	176	150

Well #1	Well #2	Well #3	Ave.	St. Dev.	% Mig #10	Well #1	Well #2
9	8	6	7	2	CD3	17	15
17	15	11	14	3	CD4	32	29
2	1	0	1	1	CD8	6	5
18	16	12	16	3	Naïve	36	32
18	16	12	16	3	Naïve CD27+	36	32
21	18	14	17	4	Group A	35	32
20	18	13	17	3	Group A CD27+	35	31
7	6	4	5	2	Group B&C	9	8
6	5	3	5	2	B&C CD27+	9	7
10	9	5	8	3	B&C CD27-	12	10

p Values to CCL21 alone	
**0.0205**	CD3
**0.0164**	CD4
**0.0175**	CD8
**0.0120**	Naïve
**0.0121**	Naïve CD27+
**0.0334**	Group A
**0.0306**	Group A CD27+
0.0969	Group B&C
0.0691	B&C CD27+
0.3735	B&C CD27-

Well #3			# 11-none	Well #1	Well #2	Well #3
27356			CD3	27450	29949	27838
23772			CD4	24459	26531	24781
1513			CD8	1362	1617	1402
17277			Naïve	17868	19334	18095
17250			Naïve CD27+	17840	19304	18067
5487			Group A	5462	5937	5536
5231			Group A CD27+	5220	5673	5290
951			Group B&C	1013	1128	1030
734			B&C CD27+	801	891	815
191			B&C CD27-	196	220	200

Well #3	Ave.	St. Dev.	% Mig #11	Well #1	Well #2	Well #3	Ave.
18	17	1	CD3	18	20	18	19
33	31	2	CD4	34	37	35	35
7	6	1	CD8	6	7	6	7
38	35	3	Naïve	39	42	39	40
38	35	3	Naïve CD27+	39	42	39	40
38	35	3	Group A	37	41	38	39
37	34	3	Group A CD27+	37	40	37	38
10	9	1	Group B&C	11	12	11	11
9	8	1	B&C CD27+	10	11	10	11
13	12	1	B&C CD27-	13	15	13	14

p Values to CCL21 alone		p Values to CCL21 alon
0.3857	CD3	0.0540
0.4493	CD4	0.0572
0.0580	CD8	0.0706
0.2302	Naïve	0.1107
0.2302	Naïve CD27+	0.1107
0.0850	Group A	**0.0173**
0.1018	Group A CD27+	**0.0195**
0.2114	Group B&C	**0.0203**
0.3486	B&C CD27+	**0.0233**
0.0599	B&C CD27-	**0.0113**

# 12-none	Well #1	Well #2	Well #3
CD3	32674	35979	28862
CD4	29014	31774	25832
CD8	1820	2151	1439
Naïve	21686	23692	19372
Naïve CD27+	21673	23677	19362
Group A	6037	6627	5356
Group A CD27+	5815	6383	5161
Group B&C	1181	1327	1013
B&C CD27+	896	1007	769
B&C CD27-	255	288	217

St. Dev.	% Mig #12	Well #1	Well #2	Well #3	Ave.	St. Dev.
1	CD3	21	24	19	21	2
2	CD4	41	45	36	40	4
1	CD8	8	10	7	8	2
2	Naïve	47	52	42	47	5
2	Naïve CD27+	47	52	42	47	5
2	Group A	41	45	37	41	4
2	Group A CD27+	41	45	36	41	4
1	Group B&C	12	14	11	12	2
1	B&C CD27+	11	13	10	11	1
1	B&C CD27-	17	19	15	17	2

e		p Values to CCL21 alone
	CD3	0.0558
	CD4	0.0544
	CD8	0.4615
	Naïve	0.0588
	Naïve CD27+	0.0584
	Group A	**0.0450**
	Group A CD27+	**0.0462**
	Group B&C	**0.0443**
	B&C CD27+	0.0587
	B&C CD27-	**0.0226**

# 13-none	Well #1	Well #2	Well #3	# 14-none
CD3	29880	31473	27520	CD3
CD4	26404	27722	24453	CD4
CD8	1887	2062	1628	CD8
Naïve	19330	20268	17943	Naïve
Naïve CD27+	19320	20256	17934	Naïve CD27+
Group A	5764	6060	5327	Group A
Group A CD27+	5534	5817	5115	Group A CD27+
Group B&C	1212	1289	1098	Group B&C
B&C CD27+	955	1015	866	B&C CD27+
B&C CD27-	231	248	208	B&C CD27-

% Mig #13	Well #1	Well #2	Well #3	Ave.	St. Dev.	% Mig #14
CD3	20	21	18	19	1	CD3
CD4	37	39	34	37	2	CD4
CD8	9	9	7	8	1	CD8
Naïve	42	44	39	42	3	Naïve
Naïve CD27+	42	44	39	42	3	Naïve CD27+
Group A	39	41	36	39	3	Group A
Group A CD27+	39	41	36	38	2	Group A CD27+
Group B&C	13	13	11	13	1	Group B&C
B&C CD27+	12	13	11	12	1	B&C CD27+
B&C CD27-	16	17	14	15	1	B&C CD27-

	p Values to CCL21 alone	
CD3	0.0742	CD3
CD4	0.0791	CD4
CD8	0.4687	CD8
Naïve	0.1165	Naïve
Naïve CD27+	0.1150	Naïve CD27+
Group A	**0.0392**	Group A
Group A CD27+	**0.0417**	Group A CD27+
Group B&C	**0.0247**	Group B&C
B&C CD27+	**0.0272**	B&C CD27+
B&C CD27-	**0.0187**	B&C CD27-

Well #1	Well #2	Well #3
29818	35317	30853
26491	31061	27351
1777	2365	1887
19478	22741	20093
19468	22727	20081
5698	6708	5888
5458	6423	5639
1183	1446	1232
924	1127	962
225	280	236

# 15-none	Well #1	Well #2
CD3	23170	21379
CD4	19962	18538
CD8	1456	1239
Naïve	14573	13572
Naïve CD27+	14567	13567
Group A	4299	3982
Group A CD27+	4150	3845
Group B&C	1000	906
B&C CD27+	774	702
B&C CD27-	204	183

Well #1	Well #2	Well #3	Ave.	St. Dev.
20	23	20	21	2
37	44	38	40	3
8	11	9	9	1
42	50	44	45	4
42	50	44	45	4
39	46	40	42	4
38	45	39	41	4
12	15	13	13	1
12	14	12	13	1
15	19	16	17	2

% Mig #15	Well #1	Well #2
CD3	15	14
CD4	28	26
CD8	7	6
Naïve	32	30
Naïve CD27+	32	30
Group A	29	27
Group A CD27+	29	27
Group B&C	10	9
B&C CD27+	10	9
B&C CD27-	14	12

p Values to CCL21 alone	
**0.0258**	CD3
**0.0260**	CD4
0.2323	CD8
**0.0336**	Naïve
**0.0333**	Naïve CD27+
**0.0167**	Group A
**0.0178**	Group A CD27+
**0.0120**	Group B&C
**0.0135**	B&C CD27+
**0.0096**	B&C CD27-

Well #3
25215
21589
1703
15716
15709
4662
4499
1108
857
227

Well #3	Ave.	St. Dev.
17	15	1
30	28	2
8	7	1
34	32	2
34	32	2
32	30	2
31	29	2
12	11	1
11	10	1
15	14	1

p Values to CCL21 alone
0.1898
0.0903
0.0866
0.0509
0.0514
0.2954
0.2790
**0.0430**
0.0605
**0.0180**
