# Supplementary material for: A humanized monoclonal antibody against the endothelial chemokine CCL21 for the diagnosis and treatment of inflammatory bowel disease
Source: PLoS One. 2021 Jul 1;16(7):e0252805. doi: 10.1371/journal.pone.0252805 (PMC8248966; doi:10.1371/journal.pone.0252805)
Supplement: S9 Fig — (PDF) [file pone.0252805.s009.pdf]

Percent migration of CD3+ CD4+

	Control	V1	V2	V3	V4	V5	V6	V7	V8	V9	V10	V11	V12	V13	V14	V15	V16
Well 1	49.5	34.1	28.6	43.6	43.6	35.7	9.2	33.6	31	29.8	37.3	36	34.7	32.7	31.8	40.3	40.9
Well 2	58.6	37.9	30.2	35.9	43	38.6	14.5	36.5	34.9	34.5	43.2	35.8	27.1	28.7	32.2	36.3	34.7
Well 3	60.2	40.1	26.9	40.7	42.9	34.8	11.2	34.7	30.7	33.9	40.8	38.7	28.7	30.5	29.7	43.7	37.3
Average	56.1	37.4	28.6	40.1	43.2	36.4	11.6	34.9	32.2	32.7	40.4	36.8	30.2	30.6	31.2	40.1	37.6
