# Supplementary material for: A humanized monoclonal antibody against the endothelial chemokine CCL21 for the diagnosis and treatment of inflammatory bowel disease
Source: PLoS One. 2021 Jul 1;16(7):e0252805. doi: 10.1371/journal.pone.0252805 (PMC8248966; doi:10.1371/journal.pone.0252805)
Supplement: S10 Fig — (PDF) [file pone.0252805.s010.pdf]

Donor 1 % CD3+ CD4+ CD8- CCR7+ CD45RA+ CD27+ Migration

	Control			100ug V6		
CCL21 (ng/mL)	Well 1	Well 2	Well 3	Well 1	Well 2	Well 3
0	0	0	0	0	0	0
100	10.1	9.4	5.5	0	1.2	0
500	28.7	25.8	29.2	2.1	0	1.6
800	36.7	34.8	33.6	5.6	4.3	2.1
1200	49.6	46.7	49.3	11	10.2	13.6
1600	46.8	50.1	48.8	14.8	10.9	15.2

Donor 2 % CD3+ CD4+ CD8- CCR7+ CD45RA+ CD27+ Migration

	Control			100ug V6		
CCL21 (ng/mL)	Well 1	Well 2	Well 3	Well 1	Well 2	Well 3
0	0	1.6	0	0	1.6	0
100	8.4	12.7	11.3	0	3.8	0
500	24.7	30.2	22.8	5.32	7.85	6.66
800	45.7	40.3	49.2	9.84	11.74	14.52
1200	60.2	64.3	61.7	19.23	22.87	24.25
1600	58.9	66.4	60.9	25.12	29.41	24.77

Donor 3 % CD3+ CD4+ CD8- CCR7+ CD45RA+ CD27+ Migration

	Control			100ug V6		
CCL21 (ng/mL)	Well 1	Well 2	Well 3	Well 1	Well 2	Well 3
0	0	0	0	0	0	0
100	8.4	2.7	6.66	0	0	0
500	17.2	15.4	11.9	2.9	3.33	1.8
800	28.4	25.8	22.4	10.9	8.1	7.06
1200	32.8	36.1	30.8	8.7	10.2	6.9
1600	44.6	46.7	40.1	14.2	16.7	12.9
